# Feeding in mixoplankton enhances phototrophy increasing bloom-induced pH changes with ocean acidification

**DOI:** 10.1093/plankt/fbad030

**Published:** 2023-07-06

**Authors:** Kevin J Flynn, Aditee Mitra

**Affiliations:** Plymouth Marine Laboratory, Prospect Place, Plymouth PL1 3Dh, UK; School of Earth and Environmental Sciences, Main Building, Park Place, Cardiff University, Cardiff CF10 3AT, UK

**Keywords:** mixoplankton, zooplankton, phytoplankton, pH, ocean acidification, CO_2_-drawdown

## Abstract

Plankton phototrophy consumes CO_2_, increasing seawater pH, while heterotrophy does the converse. Elevation of pH (>8.5) during coastal blooms becomes increasingly deleterious for plankton. Mixoplankton, which can be important bloom-formers, engage in both photoautotrophy and phagoheterotrophy; in theory, this activity could create a relatively stable pH environment for plankton growth. Using a systems biology modelling approach, we explored whether different mixoplankton functional groups could modulate the environmental pH compared to the extreme activities of phototrophic phytoplankton and heterotrophic zooplankton. Activities by most mixoplankton groups do not stabilize seawater pH. Through access to additional nutrient streams from internal recycling with phagotrophy, mixoplankton phototrophy is enhanced, elevating pH; this is especially so for constitutive and plastidic specialist non-constitutive mixoplankton. Mixoplankton blooms can exceed the size of phytoplankton blooms; the synergisms of mixoplankton physiology, accessing nutrition via phagotrophy as well as from inorganic sources, enhance or augment primary production rather than depressing it. Ocean acidification will thus enable larger coastal mixoplankton blooms to form before basification becomes detrimental. The dynamics of such bloom developments will depend on whether the mixoplankton are consuming heterotrophs and/or phototrophs and how the plankton community succession evolves.

## INTRODUCTION

Plankton are key organisms affecting life in the oceans and, via their contributions to biogeochemistry, life on Earth. Proton (H^+^) gradients (i.e. pH as -log_10_ [H^+^]) are critical features affecting the physiology of these organisms, such as enzyme activities and nutrient transport (Raven, 1980). Marine planktonic microbes have little ability to isolate themselves from external conditions and are thus directly affected by events that change seawater pH. Seawater pH is primarily controlled by the dissolved inorganic carbon (DIC) carbonate chemistry, as the equilibria between carbonate (CO_3_^—^), bicarbonate (HCO_3_^—^) and carbon dioxide (CO_2_). Changes in the partial pressure of atmospheric CO_2_ (i.e. pCO_2_) alter this chemistry via air–sea gas exchange. Atmospheric pCO_2_ has been increasing since the dawn of the industrial age; the current “business as usual” scenario projects a more than doubling of pCO_2_ from the pre-industrial level (ca. 280 ppm) to ca. 800 ppm by the end of the 21st century (CMIP6 ssp370 2100 scenario; O'Neill *et al.*, 2016; Riahi *et al.*, 2017; Meinshausen *et al.*, 2020). The dissolution of the additional atmospheric CO_2_ into the ocean increases seawater [H^+^], giving rise to “ocean acidification” (Caldeira and Wickett, 2003; Doney *et al.*, 2009).

In addition to the long-term decrease in baseline seawater pH with ocean acidification, [H^+^] varies over much shorter timescales in consequence of seasonal biological processes. The main affect is via changes in the DIC concentration through consumption of CO_2_ by photosynthesis and its release by respiration. Smaller changes in [H^+^] are associated with changes in alkalinity due to biological processes, especially by the addition or removal of NH_4_^+^, NO_3_^—^, PO_4_^—^ and Ca^++^. During primary production, consumption of CO_2_ (especially supported by consumption of NO_3_^—^ and PO_4_^—^) decreases seawater [H^+^], a process called basification. In contrast, heterotrophic activity, consuming the bloom biomass, releases CO_2_, NH_4_^+^ and PO_4_^—^, results in an increase in [H^+^]. Not only has the seawater [H^+^] increased with ocean acidification, but it has also risen above the [H^+^] of the maximum pH buffering capacity of the carbonate-chemistry system. In consequence, changes in DIC and alkalinity with biological activity are now associated with a greater initial change in [H^+^] than it did previously (Thomas *et al.*, 2007; Hofmann *et al.*, 2010; Flynn *et al.*, 2012).

The increase in average ocean [H^+^] with ocean acidification alters the starting conditions for blooms of planktonic primary producers. This initially provides more dissolved CO_2_ and, as CO_2_ is the substrate for the key enzyme of carbon fixation RuBisCO, the growth of photosynthesizing plankton is thus potentially enhanced by ocean acidification (Riebesell *et al.*, 2007; Egge *et al.*, 2009). Under nutrient-replete conditions in coastal waters, the development of extensive plankton blooms can result in an increase in the pH (decrease in [H^+^]) to levels that are lethal to plankton (Hansen, 2002; Hinga, 2002; Raven *et al.*, 2020). With ocean acidification, commencing growth at a higher [H^+^] extends the potential period of nutrient-sufficient growth before basification becomes lethal. However, changes in the buffering capacity of the seawater also see microbial plankton experiencing more rapidly changing conditions of [H^+^] (Flynn *et al.*, 2012). An individual microbial organism is affected by both the [H^+^] changes in the water column and diffusion gradients closer to the organisms themselves (Kühn and Raven, 2008; Flynn *et al.*, 2012); larger microbial plankton experience greater proximal changes in [H^+^]. In addition, changes in [H^+^] also affect aspects of behaviour and reproduction in other plankton leading to deleterious impacts on the food chain (Pedersen and Hansen, 2003; Kim *et al.*, 2013; Cripps *et al.*, 2014a, b). Such rapid significant changes in [H^+^] are far more likely in coastal waters (Borges and Gypens, 2010; Cai *et al.*, 2011), where production exploiting eutrophication sees cycles of basification (with net primary production) and acidification (with net heterotrophy; Duarte *et al.*, 2013; Nixon *et al.*, 2015).

Ocean acidification thus has the potential to radically change the pattern of succession for primary producers (Hinga, 2002; Flynn *et al.*, 2015; Wei *et al.*, 2022) and the subsequent trophic interactions that would ultimately affect fish and other top trophic consumers and thence ecosystem services. This interpretation of events assumes the traditional paradigm for marine production, centred around phytoplankton and zooplankton, with their clear contrasting activities affecting carbonate chemistry, seawater alkalinity and thence [H^+^]. However, we now appreciate that there is another group of organisms involved in plankton ecology, namely, the mixoplankton (Flynn *et al.*, 2019; Glibert and Mitra, 2022). Mixoplankton are protists that combine phytoplankton-like photosynthesis and zooplankton-like feeding activity, simultaneously and synergistically in the one organism cell. Once considered as a rather trivial ecological aside, it now appears that mixoplankton are important players in many waters, from oligotrophic gyres to eutrophic nearshore and coastal areas (Mitra *et al.*, 2023a). Protist non-diatom harmful algal bloom (HAB) species are also predominantly mixoplankton (Mitra and Flynn, 2021), and we have scant understanding of the interactions between eutrophication, ocean acidification and HABs (Wells *et al.*, 2015; Raven *et al.*, 2020).

**Fig. 1:**
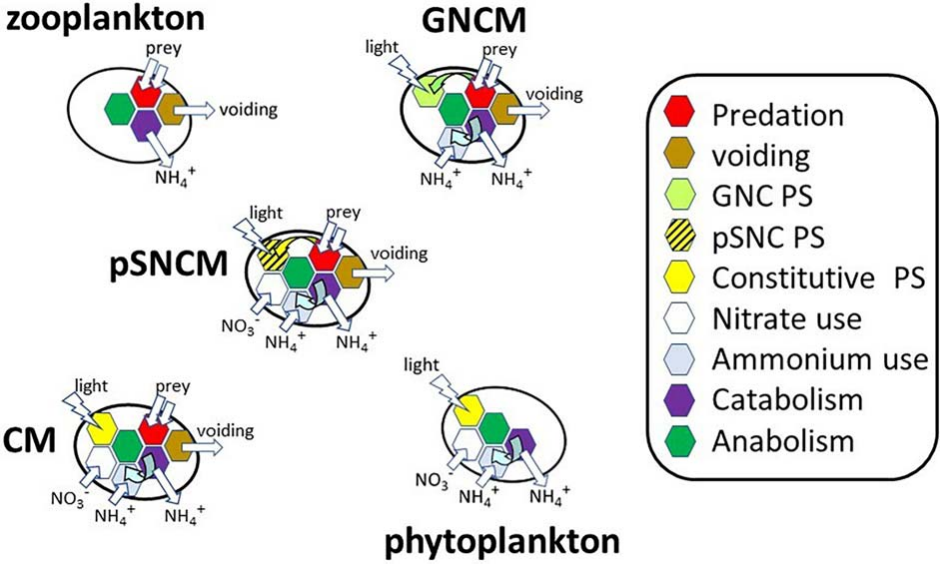
Schematic showing the different physiologies present within protist plankton types. GNCM, generalist non-constitutive mixoplankton (these acquire phototrophy from plastids derived from many prey types, but with little maintenance and control); pSNCM, plastidic specialist non-constitutive mixoplankton (these acquire phototrophy from a specific prey type, complete with some nucleic material and hence can achieve some degree of plastid maintenance and photoacclimative control); CM, constitutive mixoplankton (these have an innate ability to make plastids and have the same level of maintenance and control abilities as do phytoplankton). GNCM cannot use nitrate; pSNCM as described here can use nitrate, though not all species can do so. PS—photosynthesis.

An appreciation of the physiological differences between the protist functional groups, phytoplankton, zooplankton and mixoplankton, can be made from Fig. 1. There are different functional types of mixoplankton (Mitra *et al.*, 2016; Flynn *et al.*, 2019), distinguished by whether they possess constitutive phototrophic capabilities (constitutive mixoplankton; CM) or need to acquire this capability from their prey (non-constitutive mixoplankton; NCM). The CM have an ability to use inorganic nutrients, just like phytoplankton do, in addition to their phagotrophic capabilities. The NCM functional group includes the generalist (GNCM) and plastidic-specialist (pSNCM) forms. GNCM acquire phototrophy from many prey types, though with no capability to maintain that trait over time (thus they require frequent top-ups from phototrophic prey), and have limited (or zero, for nitrate) ability to exploit inorganic nutrient sources (Schoener and McManus, 2017; Mitra *et al.*, 2023b). The pSNCM acquire phototrophy from specific prey species or clades (though they are able to feed on many prey types for nutrition), with some measure of maintaining and acclimating their acquired photosystems and with an ability to use inorganic nutrients including nitrate (Wilkerson and Grunseich, 1990, but cf. García-Portela *et al.*, 2020).

**Fig. 2:**
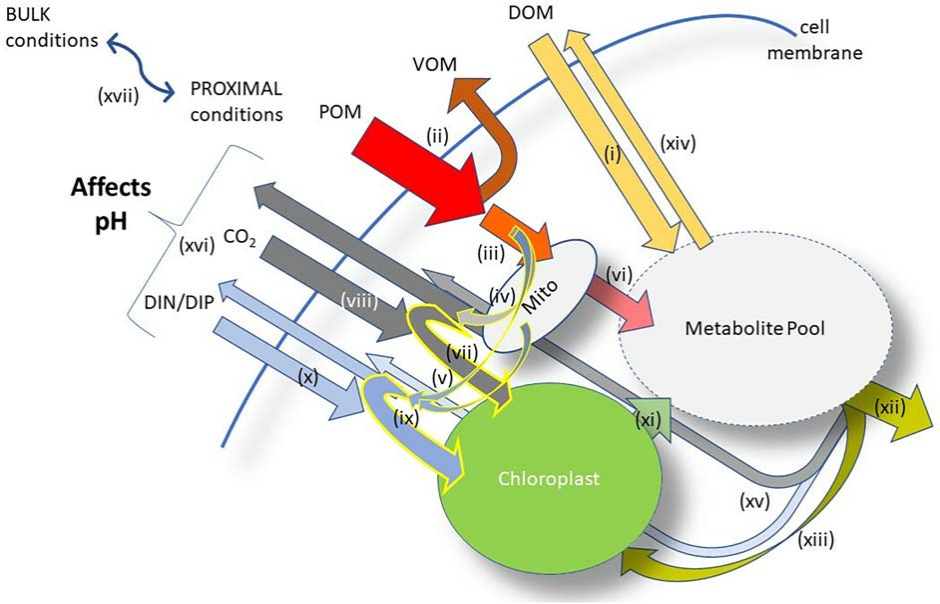
Protist plankton resource acquisition mechanisms. Not all mechanisms may be present in all protist types (see Fig. 1). Dissolved organic matter (DOM; sugars, amino acids, etc.) is taken up (i) and enters the metabolite pool; this action supports osmotrophy. Particulate organic matter (POM; such as prey) is engulfed, and a fraction (ca. 20–40%) is egested as voided organic matter (VOM) during digestion (ii). The retained fraction is broken down and a fraction (ca. 30%) is lost through specific dynamic action (SDA; iii) as CO_2_ (iv) and as dissolved inorganic nutrients (DIN, as ammonium; DIP as phosphate; v). This anabolic activity is associated with the mitochondria (Mito) and other sub-cellular compartments. The resultant remaining digestate enters the metabolite pool (vi); this activity, with (ii) and (iii), constitutes phagotrophy. The CO_2_, DIN and DIP lost through SDA contribute to meeting the CO_2_ demands for photosynthesis in chloroplasts (yellow-edged arrow, vii), with the balance of the CO_2_ demand being brought in from outside of the cell (viii). Similarly, any additional demand for DIN and/or DIP over that supplied by recycling (yellow-edged arrow, ix) is brought in from outside (x). Products from phototrophy contribute to the metabolite pool (xi). The total metabolite pool supports biomass growth (xii) including synthesis of chloroplasts (xiii). Excess metabolites are leaked (xiv), and there are additional losses of CO_2_ through respiration, with allied regeneration of DIN (as ammonium) and DIP to maintain cellular stoichiometric balance (xv). The net uptake vs release of CO_2_ and DIN, DIP (xvi), including whether DIN uptake comprises nitrate vs ammonium (ammonium being the form released), modifies seawater alkalinity and carbonate chemistry, and thence affects [H^+^] and pH. Osmotrophy (i) has an unknown impact on pH, depending on the buffering capacity of different organic chemicals, and how their assimilations may release CO_2_, DIN and DIP. The bulk water chemistry differs from the proximal conditions (next to the cell), as a function of diffusion (xvii); diffusion is slower for larger, faster-growing and/or slower-moving cells. The metabolite pool as indicated here is a general cellular pool, not located in a specific single space (i.e. it resides across the chloroplasts, mitochondria, endoplasmic reticulum, vacuoles etc.). See also Supplementary material Fig. S1.

Growth of the five different protist types (zooplankton, GNCM, pSNCM, CM and phytoplankton; Fig. 1) may be expected to affect seawater [H^+^] in different ways depending on the balance of phototrophy and phagotrophy mediated through cell physiology (Fig. 2). Heterotrophy in zooplankton is associated with losses due to inefficiency during prey assimilation (with voiding of part-digested biomass; Mitra and Flynn, 2005) and critically also in consequence of specific dynamic action (SDA). SDA sees a loss of nutrients from food that is being assimilated amounting to ca. 30% (McCue, 2006). This happens as the ingested biomass is first broken down, and the primary metabolites then reassembled into the consumer biomass, leading to the release of CO_2_, NH_4_^+^ and PO_4_^—^ into the seawater and consequential changes in DIC, alkalinity and [H^+^]. In mixoplankton, this 30% SDA loss from phagotrophy will support concurrent phototrophic demands for CO_2_ and N, P nutrition within the same organism (Fig. 2). Thus, mixoplanktonic activity sees the coupling of two contrasting physiological processes—phototrophy and phagotrophy, in the same cell (Mitra and Flynn, 2010, 2023). In terms of [H^+^], the two processes could be expected to compensate for each other within a mixoplanktonic organism.

The overarching aim of this work was to explore how the growth of different protist plankton types affect the pH of their environment. Our hypothesis was that mixoplankton may be advantaged by their dual physiological activities providing them with a relatively stable [H^+^] environment compared to the phytoplankton or zooplankton counterparts.

## METHODS

To explore how protist planktonic activities may affect [H^+^] in their growth environment, this work compared the behaviour of the five principle protist plankton functional types with respect to how their growth physiologies affect [H^+^] and how those effects may be impacted by growth commencing at different (pre-industrial vs future) atmospheric pCO_2_. The protist functional types considered were, at the extremes of the trophic spectrum, the phagotrophic zooplankton and the phototrophic phytoplankton and three types of mixoplankton—generalist and plastidic-specialist NCM (GNCM and pSNCM, respectively) and CM (Fig. 1). The physiological interactions in these different protists were explored using a model that reproduces details of protist physiology that also affect carbonate chemistry, alkalinity and [H^+^] resulting from the uptake and/or regeneration (release) of ammonium, nitrate and phosphate (Fig. 2).

The numeric (simulation) modelling approach that we have used exploited protist plankton descriptions developed previously (Flynn and Mitra, 2009; Flynn, 2021), which have been used in various works (Flynn *et al.*, 2015; Lin *et al.*, 2018; Leles *et al.*, 2021; Li *et al.*, 2022; Mitra and Flynn, 2023). Functional equations are provided in Supplementary material. The maximum protist growth rate was set at 0.693 d^−1^ (i.e. a doubling per day) at 15°C, with the organism configured as a motile cell of 20 μm equivalent spherical diameter (ESD). This combination of growth rate and cell size represents a compromise across the parameter ranges for these planktonic protists, so as to minimize the number of variables. For the mixoplankton, the controls of phototrophy and phagotrophy were, by default, configured as being modulated by the same level of cell nutrient status. Investigations were also conducted in which one or other of these trophic mechanisms was de-repressed (enabled) at higher levels of stress (see Supplementary material, Figs S1 and S2, Tables S1–S4).

Protist plankton growth was simulated with light provided in a light:dark cycle (0.7:0.3) at a photon flux density (PFD) of 500 μmol m^−2^ s^−1^, which saturated photosynthesis. Inorganic nutrients were supplied at initial concentrations of ammonium (10 μM), nitrate (10 μM) and phosphate (1.25 μM), providing dissolved inorganic nutrient (inorganic N, inorganic P; DIN, DIP, respectively) at the Redfield ratio. In addition, non-growing prey (of 5 μm ESD) were supplied; these were of Redfield C:N:P such that the N and P content were the same as that of the inorganic nutrients (i.e. with N and P at 20 μg atom N L^−1^ and 1.25 μg atom P L^−1^) and hence the initial prey biomass C abundance was 132.5 μg atom L^−1^. These concentrations and abundances were used so as to be consistent with near-shore waters while not being sufficiently high that phototrophic growth would draw down DIC to levels that could significantly restrict photosynthesis (Clark and Flynn, 2000). The prey also had associated with them photosystems (described in terms of Chl and chloroplast biomass) to provide the materials required to support acquired phototrophy by GNCM and pSNCM protist configurations. Non-growing prey were provided so as to simplify interpretation of the results. Test scenarios also included investigations of the impact on CM growth of doubling prey abundance, providing prey of poor quality (halved N:C and P:C) and/or greater quantity, or providing a lower irradiance (PFD 50 μmol m^−2^ s^−1^).

This work investigated the role that the individual organism has on seawater [H^+^] through changes in DIC and nutrients. Accordingly, for such an autecological study, the model described a batch-culture setup for each of the protist functional types, in a water body initially at equilibrium with a stated atmospheric pCO_2_ but thereafter considered to be closed to gas exchange. Values of pCO_2_ (atm) were used for that equilibrium corresponding to the early industrial age (300 atm) or the end-of-21st-century ssp245 projection of 600 atm, which assumes the medium projection of future greenhouse gas emissions (O'Neill *et al.*, 2016; Meinshausen *et al.*, 2020). Impacts of these pCO_2_ values for the seawater carbonate system were calculated at 15°C and salinity 35. The carbonate chemistry description follows that we have used before (Flynn *et al.*, 2012; see Supplementary material); this gives results consistent with that given by the CO2sys calculator (Lewis *et al.*, 1998) and has been shown to closely fit experimental data for plankton growth (Flynn *et al.*, 2015). To place the biological physiologies in context, in Supplementary material Fig. S3, we show the changes in [H^+^] that occur in consequence of photosynthesis or respiration purely in terms of DIC, and with additional alkalinity changes with phototrophy using ammonium or nitrate together with phosphate, or with heterotrophic regeneration of ammonium and phosphate.

Emphasis in the results is given to [H^+^], as this is what organisms experience (pH being −log_10_ [H^+^]). Near-cell, proximal, [H^+^] values were computed from physiological rates into cells of a stated ESD and bulk water [H^+^] (Flynn *et al.*, 2012, 2016). We also considered the impact upon proximal [H^+^] of different cell size, assuming the same physiological rates and using the same prey sizes, although larger protists encounter more prey, so their grazing rates were higher. Because we do not know how [H^+^] interacts with the physiology of mixoplankton, the carbonate chemistry model operated downstream of the biological model; there was no interaction term from [H^+^] to physiology similar to that we have configured against empirical data and used before for phytoplankton (Flynn *et al.*, 2015). As there was also no effective feedback from changes in carbonate chemistry to physiology (DIC being always far in excess and thence not limiting for photosynthesis), the nutrient and biomass dynamics were the same irrespective of the pCO_2_. In consequence, the plots show the same physiological changes irrespective of the initial pCO_2_ but reveal the consequences of those changes upon the DIC, [H^+^] and pH for the different pCO_2_ scenarios.

## RESULTS

Plots in Supplementary material are numbered in the style Fig. Sx and Table Sx.

### Comparisons between protist plankton functional types

**Fig. 3:**
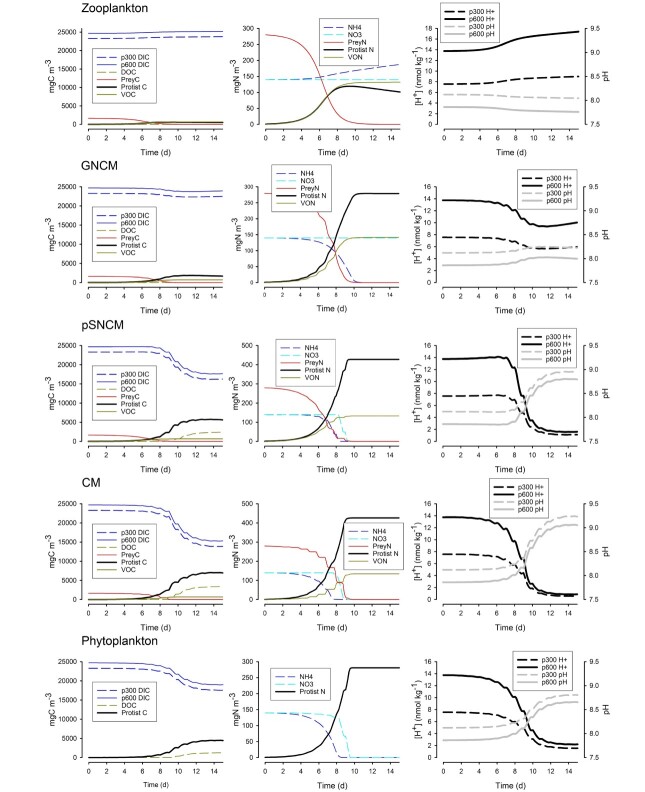
Changes in C, N and [H^+^] during the simulated growth of different protist plankton. See Fig. 1 for schematics of the physiological configurations of these organisms. DIC—dissolved inorganic C (shown attributed to either systems starting with air-sea CO_2_ equilibrium of pCO_2_ 300 or 600 atm, as p300 or p600, respectively); DOC—dissolved organic C; VOC, VON—voided organic C or N as micro-faeces; NH4—ammonium; NO3—nitrate. Corresponding changes in P are shown in Supplementary material Fig. S4. [H^+^] is shown for the bulk water; the proximal [H^+^] for a portion of the simulation is shown in Supplementary material Fig. S5. See also Supplementary material Table S5.

**Fig. 4:**
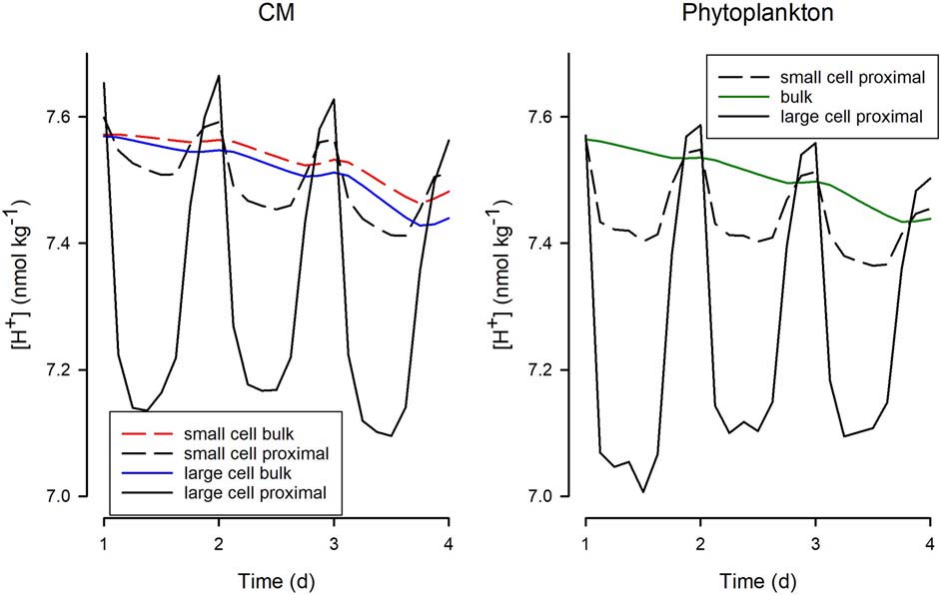
Changes in bulk and near-cell (proximal) [H^+^] for CM and phytoplankton. These are shown for the 300 atm pCO_2_ scenarios. The “small” cell versions are the same as shown in Fig. 3 and Supplementary material Figs S4 and S5, with an average cell ESD of 20 μm. The “large” cells have an average ESD of 250 μm. Growth is simulated in a light:dark cycle, with the latter 0.3 of each day in darkness, hence the increase in [H^+^] at that time period, as respiration exceeded photosynthesis.

**Fig. 5:**
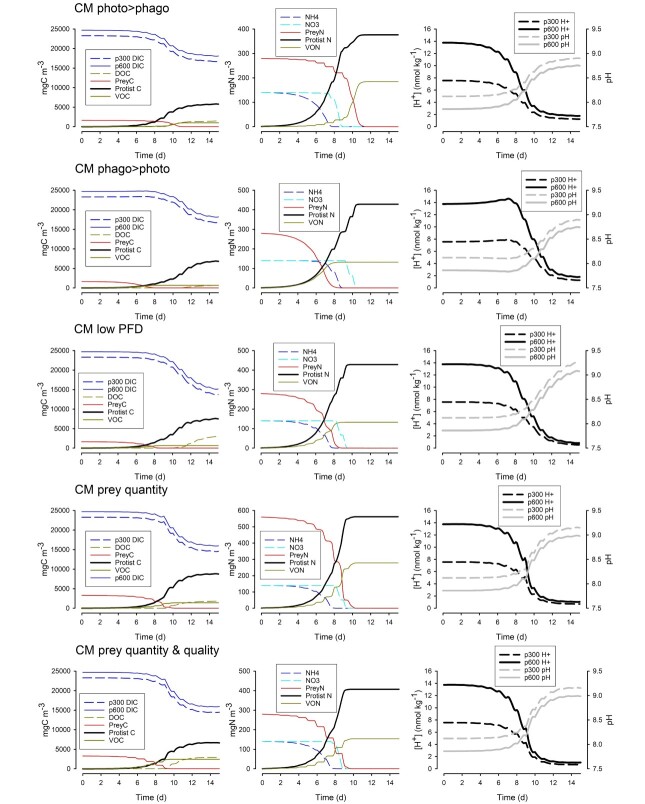
Effects of CM growth under different configurations and scenarios. “CM photo>phago”—with priority to phototrophy; “CM phago>photo”—with priority to phagotrophy; “CM low PFD”—light provided as 50, rather than the default, 500 μmol m^−2^ s^−1^; “CM prey quantity”—with twice the default prey abundance; “CM prey quantity & quality”—twice the default prey abundance but with prey of half the N:C and P:C. Corresponding changes in P are shown in Supplementary material Fig. S4. [H^+^] is shown for the bulk water; the proximal [H^+^] for a portion of the simulation is shown in Supplementary material Fig. S5. See also Supplementary material Table S5.

Results from the simulations (Figs 3–5) show changes in the C and N components of the system, and in acidity (as both [H^+^] and pH), during batch growth of each of the different protist functional types in a system with no gas exchange; P components are shown in Fig. S4. In all instances, as there was no simulated feedback between acidity and physiology, the plots for C, N and P dynamics are identical in both the 300- and 600-atm pCO_2_ scenarios, with the exception (as plotted) for dissolved inorganic C (DIC). A summary of the end-of-simulation results is given in Table S5.

Zooplankton growth (Figs 3 and S4) caused an increase in [H^+^] as the protist consumed prey, voided a fraction and respired DIC while concurrently regenerating ammonium and phosphate and leaking dissolved organic C (DOC), which were subsequently recovered. The increase in [H^+^] during this process was greater in the 600-atm pCO_2_ scenario because the buffering capacity of seawater was lower than in the 300-atm pCO_2_ scenario. In contrast to the zooplankton, the GNCM and pSNCM are also phototrophic, acquiring that phototrophy from prey ingestion. GNCM have an ability to use ammonium (as implemented here) but cannot use nitrate; pSNCM can use both N-sources. The acquired phototrophy in GNCM and pSNCM compensated for the losses of C, N and P that are seen in the zooplankton (Figs 3 and S4). There was now a distinct diel pattern to the concentrations of C,N,P components and in [H^+^], which are discernible from the stepped form of the plot data (see also Fig. S5). While prey were available, the ability of GNCM to perform photosynthesis via acquired phototrophy consumed CO_2_ and hence [H^+^] decreased; that change in [H^+^] was much greater in the 600-pCO_2_ scenario. However, on exhaustion of the prey (after 10 days), with the fading of the phototrophic potential of this mixoplankton type, respiration became increasingly dominant and there was then a net increase in heterotrophy (akin to that in zooplankton), resulting in the [H^+^] increasing. The net production of DOC was small (Table S5).

In contrast to GNCM, the pSNCM configuration has an enhanced acquired phototrophic potential maintaining their acquired photosystems better, so they last longer, and they can also use nitrate (Fig. 1). Thus, the matching of predation and phototrophy (Figs 3 and S4) for pSNCM initially suppressed the diel change in [H^+^] (Fig. S5); beyond day 8 (as the prey abundance declined) phototrophy became increasingly dominant causing clear diel step changes in [H^+^]. Production of DOC continued after exhaustion of the prey and consumption of inorganic nutrients, with phototrophy continuing until the cells attained their maximum C:N stoichiometry.

The CM configuration (Figs 3 and S4) performed in a similar way to the pSNCM, being able to use ammonium and nitrate. However, as phototrophy was no longer acquired from predation, the ability to fix CO_2_ did not decay on exhaustion of the prey. Predation showed a stronger diel signal, as did [H^+^] (Fig. S4); in the default CM configuration phagotrophy was controlled by nutritional status in a similar way to phototrophy (Fig. S2) — see below for results with other configurations. The phytoplankton configuration (Figs 3 and S4), displayed photosynthesis with the use of ammonium and nitrate, but with no predation (the prey biomasses are not shown in the plots). While there was a clear diel cycle of growth and of a diel oscillation of near-cell [H^+^] (Fig. S5), [H^+^] did not decline as far as it did for CM, being more similar to that for pSNCM (Table S5).

Figure 4 shows just the first few days of the CM and phytoplankton plots presented in full within Fig. 3. Also shown is an illustration of the consequences for running the same simulated rate dynamics but with protist cells of different sizes (prey were of the same size). As ion gradients are greater with larger cells, because the diffusion distances are of greater consequence, changes in the proximal [H^+^] are more pronounced than with smaller cells assuming similar physiological rates. There are different bulk cell plots for small and large CM (Fig. 4) because larger cells encountered more prey. There was, however, no effective difference in inorganic nutrient acquisition, hence the bulk nutrient concentration values for the phytoplankton cells were the same. There was a rather marginal overall difference between the organisms (CM vs phytoplankton) when considering the diel cycle of [H^+^].

### Variation in the CM responses

In Fig. 5 (and Fig. S4 for the allied P dynamics) are shown simulations using alternative configurations of the CM and grown in different operational scenarios. Between them, these different responses provide an insight for how different mixoplankton activities (here, specifically CM) and ecological scenarios may affect production and changes in [H^+^]. The default CM configuration (as used for Fig. 3) placed equal priority on phototrophy versus phagotrophy (see Fig. S2 for an explanation of how repression control was modulated in the model). The configuration prioritizing phototrophy (“CM photo>phago” in Fig. 5) behaved broadly similar to the default, though it produced less DOC (ca. 50% of production in the default), and thus ultimately, [H^+^] was not so low (pH did not rise so high; Table S5). In contrast, the configuration prioritizing phagotrophy (“CM phago>photo” in Fig. 5) showed an initial increase in [H^+^] due to higher net heterotrophy before increasing phototrophy decreased the [H^+^]. This was similar to the behaviour of the pSNCM in Fig. 3. Over the period of the simulation, this “CM phago>photo” configuration also produced less DOC (ca. 25% of the default; Table S5). The alternative CM configurations gave different initial proximal diel cycles in [H^+^], with “CM photo>phago” being most similar to phytoplankton (Fig. S5). Running the default CM configuration (which places equal emphasis on phototrophy and phagotrophy) at limiting light (“CM low PFD” in Fig. 5 vs CM in Fig. 3) gives a similar end result to that seen when prioritizing phagotrophy at high light (“CM phago>photo” in Fig. 5; Table S5).

Changing prey quantity and quality affected the contribution that phagotrophy made to mixoplankton production and thence affected changes in [H^+^]. Increasing the prey quantity, with the same good quality (“CM prey quantity” in Fig. 5 vs CM in Fig. 3) did not result in more C-fixation overall (and DOC decreased to ca. 60% of default; Table S5), but there was more voided material (more than doubled, in line with the higher ingestion). This reflects a change in the balance of photo- vs phago-trophy, being skewed more towards the latter because C that would otherwise be released as DOC was directed to aid assimilation of prey N and P released by SDA. Increasing the prey quantity, in terms of C, while simultaneously halving the quality (thus the availability of prey-N and prey-P remained the same; “CM prey quantity & quality” in Figs 5 and S4), resulted in even more C being voided (approaching four times that in the default, Fig. 3). More DOC was released (50% greater) in the poor prey quality situation compared with that from “CM prey quantity,” though this was less than in the default (Fig. 3, Table S5); this reflected the poorer support of mixoplankton growth by ingestion of low-value prey. Consistent with this, the diel variation in [H^+^] was greater for the low-quality prey scenario, as growth of the CM in that scenario was more dependent on phototrophy (Fig. S5).

## DISCUSSION

Much research has been conducted on the interactions between seawater [H^+^] (often referenced to “ocean acidification”) and phytoplankton (Hinga, 2002; Hansen *et al.*, 2007; Egge *et al.*, 2009; Kim *et al.*, 2013; Bach *et al.*, 2017; Hyun *et al.*, 2020; Raven and Beardall, 2021) and rather less on zooplankton (Pedersen and Hansen, 2003; Cripps *et al.*, 2014a, b; Garzke *et al.*, 2016; Jin *et al.*, 2020; Bhuiyan *et al.*, 2022). However, we have hitherto had little or no appreciation of the interactions between mixoplankton and [H^+^] other than coincidentally in works where species belonging to this functional group have been de facto considered as either phytoplankton or zooplankton (Smith and Hansen, 2007; Kim *et al.*, 2013).

Set against increases in the baseline seawater [H^+^], biological activity during bloom development and subsequent consumption can (depending on the nutrient loading) give rise to significant cyclical changes in [H^+^] (Flynn *et al.*, 2012). The activities of phytoplankton (Fig. 3) decrease seawater [H^+^] (pH increases), while the activities of heterotrophs such as zooplankton (Fig. 3) make the seawater more acidic. The dynamics of these events have been changed by ocean acidification because of the decreased buffering capacity of the carbonate system when starting at a higher [H^+^] (Supplementary material Fig. S3; Thomas *et al.*, 2007; Hofmann *et al.*, 2010). Phototrophy commencing in more acidic seawater causes basification to occur over the span of greatest buffering capacity of the carbonate system (Flynn *et al.*, 2012). Extremes of seawater [H^+^] (high or low) cause stress in plankton, and set against the increase in baseline [H^+^] and bloom-generated basification, nutrient stress in waters of different nutrient loading is expected to affect plankton succession with ocean acidification (Flynn *et al.*, 2015).

From the above, one may expect the growth of mixoplankton, protists that couple phagotrophy and phototrophy (Mitra *et al.*, 2014; Flynn *et al.*, 2019), to not promote such extreme changes in acidity as do the activities of either the non-phototrophic zooplankton or the non-phagotrophic phytoplankton. Further, one may expect any moderation of changes in acidity to be of greater consequence at a higher pCO_2_ as changes in [H^+^] are greater per unit of physiological activity (Supplementary material Fig. S3). This potential of mixoplankton engaging in phagoheterotrophy to modulate the [H^+^] of their environment against the increases in seawater [H^+^] caused by phototrophy, has parallels with the possible role of calcification in planktonic coccolithophorids, a process that releases CO_2_ and increases [H^+^] (Flynn *et al.*, 2016). This concept formed the basis of our original hypothesis that mixoplankton growth will have less impact on [H^+^] during the growth of blooms, and thence, blooms of mixoplankton are less likely to lead to damaging extremes of [H^+^]. Our results do not support this hypothesis.

### Balancing photoautotrophy and phagoheterotrophy within mixoplankton

In the simulations, the closest we see to the balance point between phototrophy and phagotrophy, to attain net zero changes in [H^+^], is for GNCM (Fig. 3). These are protists that perform acquired phototrophy that primarily compensates for respiration and regeneration losses with SDA. Most of the nutrients are internally recycled, nitrate cannot be used (Schoener and McManus, 2017) and the restricted longevity of the acquired phototrophy (Stoecker *et al.*, 1988; Flynn and Hansen, 2013) limits net CO_2_ fixation once the prey are eliminated (Fig. 3). The pSNCM, although also acquiring phototrophy (though from its specific prey: Johnson *et al.*, 2006; Park *et al.*, 2008), behaves in a more similar way to the CM (Fig. 3). This is because pSNCM can maintain the competency of their photosystems long beyond the period of acquiring the phototrophy from their prey (Stoecker *et al.*, 2009; Hansen *et al.*, 2013), enabling continued bloom development using inorganic nutrients. Although the pSCNM does not draw down as much CO_2_ as the CM, in the simulations, it still exceeds the drawdown by the phytoplankton (Supplementary material Table S5). This is because both of these mixoplankton types exploit nutrients recycled internally during the assimilation of prey biomass to support additional phototrophy; the phytoplankton can only access the inorganic nutrients available in the external environment. This is not to say, however, that at a given point in time, phototrophy is necessarily dominant; the early stages of the pSNCM simulation showed a close matching of phototrophy and phagotrophy (Supplementary material Fig. S5) as, in the simulation, this phase of growth was associated with the pSNCM photoacclimating from having a depressed phototrophy. The GNCM, in contrast, rapidly inherited a fully functioning phototrophic potential from ingesting its phototrophic prey and showed a diel cycle of proximal [H^+^] in consequence (Supplementary material Fig. S5), although this ability faded rapidly on prey exhaustion (Fig. 3).

We explored the consequences of changing the priorities of phototrophy and phagotrophy and of the availability of light or prey of different quantity and quality in CM (Fig. 5). While phytoplankton and zooplankton growths are sensitive to light and to prey quantity/quality, respectively, mixoplankton have the potential to compensate for shortfalls in either phototrophy or phagotrophy. However, not only does this affect their growth and changes to [H^+^], but the consequences of rebalancing nutrition for the release of DOC and POC (voided material, as VOC, VON and VOP in the simulations) can be significant (Supplementary material Table S5).

To provide additional context to these considerations, we estimated the likely balance point for zero net change in DIC associated with phagoheterotrophy versus photoautotrophy in mixoplankton. This takes into account prey assimilation efficiencies (AE), SDA during digestion and re-assimilation, other respiration activities and DOC leakages. The effect of AE, which sees a proportion of ingested prey (1-AE) voided (Fig. 2), is itself of no consequence for seawater acidity, though heterotrophic degradation of the micro-faeces acidifies the water (Nixon *et al.*, 2015). The action of SDA in a zooplankton releases CO_2_, phosphate and ammonium; the first two increases acidity, but ammonium release increases alkalinity thus decreasing the rise in [H^+^] (Supplementary material Fig. S3). However, those same physiological events occurring within a mixoplankton sees these nutrients made available in close proximity to phototrophic systems (within the same cell) that will reassimilate them internally (Fig. 2); these chemicals are thus not released to the environment, and there are no SDA-related changes in [H^+^].

According to our analysis (Supplementary discussion calculations), the balance–point ratio between phagotrophy and phototrophy to achieve a zero change in [H^+^] occurs at approximately {3× C brought in by phagotrophy}: {1× gross C-fixation}. This ratio is attained with a C-specific ingestion rate of ca. 1.3× the mixoplankton growth rate. This ingestion rate is much lower than that required to support an equal growth rate of a purely phagotrophic zooplankton (which is ca. 1.9× the zooplankton growth rate). As a first approximation, assuming a mixoplankton size of 20 μm diameter and prey of 5 μm with similar C:N:P stoichiometries and C-biovolume densities and a growth rate of 0.693 day^−1^, the ingestion rate to balance phagotrophy and phototrophy is ca. 1 prey item every 15 min. With a combination of a 10 μm CM and a 1 μm prey, the ingestion rate is ca. 1 prey min^−1^. While maximum grazing rates by mixoplankton are highly variable (Jeong *et al.*, 2010; Yoo *et al.*, 2017), these ingestion rates to balance phototrophy and phagotrophy are extremely high. The implication is that mixoplankton will most typically be net primary producers and likely strong net contributors to basification (decrease in [H^+^]). The role of phagotrophy in mixoplankton appears to be to obtain nutrients rather than C, although the significance of C increases at limiting irradiance (Mitra and Flynn, 2023).

### Proximal pH changes

For non-motile plankton, in non-turbulent water, there is a potential for a significant proximal (cell-surface) change in [H^+^] as the cells go through diel cycles of net consumption or release of CO_2_ within the light–dark cycle. This has been shown with *in silico* studies (Wolf-Gladrow and Riebesell, 1997; Flynn *et al.*, 2012) and using microprobes with cultured organisms (Kühn and Raven, 2008; Chrachri *et al.*, 2018). The proximal cycle of [H^+^] around the bulk water signal is stronger with larger cells and with higher physiological rates (Fig. 4) because of the magnitude of the diffusion gradients. Motility, seen in most mixoplankton other than species such as the endosymbiotic rhizarians (not simulated here), greatly decreases the diffusion gradients and hence brings the proximal [H^+^] values closer to those in the bulk water (Flynn *et al.*, 2012). In the simulations (Figs 3 and 4), the default CM showed only slightly different patterns of diel cycling of [H^+^] to similarly sized and equally motile phytoplankton. There is no evidence of a significant homeostatic advantage for [H^+^] in concurrent phagotrophy with phototrophy, although some configurations and scenarios show periods of concurrency that stabilize changes in [H^+^] (Supplementary material Fig. S5). In the simulations, most predation occurred in the light phase, an emergent behaviour of the model consistent with observations of real mixoplankton (Adolf *et al.*, 2003; Stoecker *et al.*, 2017, but cf. Caron *et al.*, 1990; Rottberger *et al.*, 2013; McKie-Krisberg *et al.*, 2015). In nature, however, diel vertical migration by mixoplankton, as exemplified by phototrophic flagellates typically between surface waters during the day and more resource-rich waters at depth during the night (Kamykowski, 1981; MacIntyre *et al.*, 1997), would enforce a more extreme division between phototrophy and phagotrophy and of encounters with extremes of [H^+^] (lower in surface waters during intense primary production and higher at depth with net heterotrophy).

### Varying synergisms between phototrophy and phagotrophy

Conceptual models of mixoplankton see phagotrophy as either a regular and perhaps even dominant trophic mode, or conversely primarily expressed only when phototrophy is unable to meet demands for the support of growth (Jones, 1997; Stoecker, 1998). However, the trophic modes operate across a continuum that, rather than representing trait trade-offs (Mitra *et al.*, 2023c), reflects synergism between what at first sight appear as conflicting physiological processes. The default CM model configuration (Fig. 3) set phagotrophy and phototrophy as controlled equally by the cellular nutritional status (Supplementary material Fig. S2). Setting phototrophy as the first to be de-repressed as a response to a low nutrient status, or conversely with phagotrophy as first (Fig. 5), had different temporal implications on changes in [H^+^] and ultimately on its end point (see Supplementary material Table S5 and Fig. S5 for diel proximal [H^+^] cycles). Light availability also affects the balance of phototrophy and phagotrophy and especially the relative importance of the contribution of prey-C to mixoplankton growth (Mitra and Flynn, 2023).

Ultimately, photic-zone light limitation slows rather than caps the potential for growth. For phototrophs, the extent of growth is capped by inorganic nutrients, but for mixoplankton, nutrients are also obtained directly from phagotrophy through assimilation of prey biomass and indirectly via the recovery of inorganic nutrients supporting additional phototrophy (Fig. 2). As with all predator–prey activity (e.g. for copepods, Tirelli and Mayzaud, 2005), the quality and quantity of the feed have important consequences for the mixoplankton consumer and thence for [H^+^]. Poor quality food, reflected here by a halving of the N and P content of the prey (“CM prey quantity & quality” in Fig. 5), decreases the efficiency of assimilation into the mixoplankton; not only are more of the ingested materials released (as per stoichiometric ecology), they are also lost as a more general consequence of an inferior diet (Mitra and Flynn, 2005). High food abundances are also typically handled less efficiently (Mitra and Flynn, 2007), with more material being voided. While such voiding is more efficient for the growth of the individual consumer (because resources are not wasted digesting decreasingly labile ingestate), it is less efficient for trophic transfer at the population level. Compared to zooplankton predators, mixoplankton are unique in that they have additional scope to reprocess food while also having additional overflow routes. Here, with poorer-quality feed (Fig. 5 vs Fig. 3), the release of DOC and VOC increased, with a poor CM biomass yield (Supplementary material Table S5). Experiments where prey of different nutritional quality have been fed to mixoplankton (Lundgren *et al.*, 2016; Lin *et al.*, 2018) show the emergent complexity of the interactions.

Coupled with mixoplanktonic activity, as a consequence of C entering the low molecular metabolite pool via photosynthesis and predation (Fig. 2), the mixoplankton display an elevated production of DOC (Supplementary material Table S5). The supply of nutrients from feeding, through internal cycling, supports phototrophy, while the enhanced leakage of DOC that will draw down additional CO_2_ increases basification. This release of DOC is typical of phototrophic plankton (Biddanda and Benner, 1997; Wetz and Wheeler, 2007), but from mixoplankton, there is also a production of VOC (and VON and VOP), which comprises material that operationally for chemical analysis is “dissolved” (most being <0.2 μm, which is the filter pore size for screening prior to analysis of dissolved organic matter, DOM). Most harmful algal bloom species are mixoplankton (Burkholder *et al.*, 2008; Mitra and Flynn, 2021), and these produce secondary metabolites (recognized as toxins). This raises the possibility that a proportion of these voided and leaked materials will be at least partially resistant to degradation, thus contributing to material entering the microbial carbon pump (Jiao and Azam, 2011; Glibert and Mitra, 2022). Some mixoplankton also actively release DOC to aid their grazing activity, not only in the form of lytic enzymes (Fistarol *et al.*, 2003; Manning and La Claire, 2010) but also as mucus traps that are discarded when they become clogged (Larsson *et al.*, 2022). Discarded mucus traps contribute to the sinking of DOC as polysaccharide aggregates (Engel *et al.*, 2004) as a direct consequence of primary production (Engel, 2002). During mixoplankton bloom development, the additional fixation of CO_2_ evidenced as accumulated particulate and dissolved organics will decrease [H^+^]—this is seen in the simulations (Figs 3 and 5). Subsequent degradation of this organic material by bacteria would increase [H^+^] and simultaneously consume O_2_. Though much of those processes may occur in the weeks beyond the mixoplankton bloom, there is also scope for a sudden dramatic change in the system due to the surface accumulation (with diel vertical migration) of mixoplankton. At high light and high temperature, those mixoplankton could generate high rates of photosynthesis causing extreme local basification that ultimately kills the mixoplankton and thence terminates the bloom. Conversely, a change in the weather that significantly decreases sunlight may drive the mixoplankton themselves into net heterotrophy, aided by the bacterial degradation of DOC and VOC, with consequential rapid decreases in O_2_ levels and increase in [H^+^]. The presence of both phototrophy and phagotrophy in the one mixoplankton cell provides, in one organism, population ecological processes that in traditionally conceptualized phytoplankton–zooplankton–bacteria scenarios occur over different time frames.

### Further ecological considerations

Whether the action of a particular organism, or a functional group, contributes to a significant change in bulk seawater [H^+^] depends on that organism’s growth rate and behaviour (such as diel vertical migration), the activities and dominance of the other members of the community and various abiotic factors (notably the nutrient loading, temperature, pH-buffering capacity of the water, CO_2_ gas exchange rate and mixing with adjoining water bodies). Differential effects of ocean acidification on plankton are expected to affect succession and productivity (Wei *et al.*, 2022). In consequence, the relative activities of members of the plankton will affect whether or not the system displays a net positive primary production that consumes CO_2_ and cause basification.

Above all, the nutrient loading of the water column is of importance. Low nutrient levels, such as those in oligotrophic waters, could not support sufficient CO_2_-drawdown to cause significant basification. The simulations shown here exploited 20 μg atoms N of DIN and/or of prey-N. Flynn *et al.* (2012) consider ocean acidification scenarios for phytoplankton in waters of 5 μg atom N, in which bulk water changes are minor, though proximal changes for large microplankton could be significant. Mitra and Flynn (2023) considered mixoplankton phototrophy vs phagotrophy with resource loads of <2.5 μg atoms N, showing the significance of even very low ingestion rates of bacteria into small mixoplankton of the type reported by Zubkov and Tarran (2008). The collective activity of plankton at such low nutrient loadings would have little effect on [H^+^], and, from Fig. 4, we can see that the proximal changes in acidity would be similar to those in phytoplankton of the same size and growth rate (see Flynn *et al.*, 2012). Oceanic mixoplankton will thus experience similar [H^+^] to their phytoplankton comparators, and the average [H^+^] will largely mirror that of the photic zone average. In coastal waters, where mixoplankton blooms can also form harmful growths, results from our simulations are of importance for community ecology.

Our simulations reveal that phagotrophy does not compensate for phototrophy with respect to changes in [H^+^]. This is because phagotrophy brings resources into the mixoplankton that promote phototrophy. Feeding does not therefore compensate or diminish the phototrophy that causes basification (decrease in [H^+^]) as may be assumed from considering this form of mixotrophy as simply an additive event (Mitra and Flynn, 2010). In consequence, [H^+^] can decrease (pH increases) just as much during growth by a mixoplankton as it does for a phytoplankton (Figs 3 and 4). Indeed, the extent of basification can potentially be greater through the ability of the mixoplankton to exploit additional nutrient streams (i.e. via the consumption of prey). In essence, enhanced primary production is enabled in mixoplankton by the short-circuiting of producer–consumer interactions (traditionally viewed as that between phytoplankton, bacteria and zooplankton) within the one cell (Fig. 2). The ecological consequences are complex and will take additional research effort to fully appreciate.

The trophic dynamics of the whole ecosystem, and of the physiological priorities between phototrophy and phagotrophy (Fig. 5), will all impact the temporal changes in DIC and thence of [H^+^]. To study this further requires the building of a comprehensive simulation model of the planktonic system, including descriptions of different types of mixotrophy exploiting different resource pools. The availability of light alone, a driver that is highly variable and one that is for the individual cell negatively related to biomass growth because of community self-shading, has important consequences for the balance of phototrophy and phagotrophy (Fig. 5; Mitra and Flynn, 2023). It is also noteworthy that phagotrophy may provide the mixoplankton with the means to exploit and contain their competitors. Whether those competitors, as prey items, are heterotrophic or phototrophic will also have an effect on [H^+^], as will the timing of the consumptions and inter-conversions between inorganic, dissolved organic and biomass-bound nutrients.

## CONCLUSION

Our work shows that mixoplankton have the potential for an enhanced contribution to primary production, causing greater seawater basification, than that promoted by similarly configured phytoplankton in the same inorganic nutrient regime. It appears certain that ocean acidification will enable an enhanced potential for mixoplankton bloom development before lethal high pH levels are attained even though photoautotrophy and phagoheterotrophy do not compensate in terms of [H^+^] consumption/production. Mixoplankton can achieve this potential through exploitation of additional nutrient streams available via phagotrophy. The implication is that the status of eutrophication, invariably associated with elevated levels of inorganic nutrients (Ferreira *et al.*, 2010), would benefit from inclusion of the organic nutrients that support growth of feed for mixoplankton. We also need an understanding of the [H^+^] sensitivity of different mixoplankton, and of their prey, similar to that relating nutrient status of phytoplankton to [H^+^]; these sensitivities affect succession (Hinga, 2002; Flynn *et al.*, 2015). Primary production, affecting [H^+^], also flows to production of dissolved organics. The routine determination of DOM during studies of mixoplankton would be of value to ascertain the role that these organisms have in the microbial carbon pump, noting also that many mixoplankton consume bacteria (Zubkov and Tarran, 2008; Mitra *et al.*, 2023b).

## Supplementary Material

MixOA_SM_R3_fbad030

## Data Availability

The model description, configuration and data that support the findings of this study are available in the Supplementary data associated with this article.
