# Supplementary material for: Feeding in mixoplankton enhances phototrophy increasing bloom-induced pH changes with ocean acidification
Source: J Plankton Res. 2023 Jul 6;45(4):636–51. doi: 10.1093/plankt/fbad030 (PMC10361812; doi:10.1093/plankt/fbad030)
Supplement: MixOA_SM_R3_fbad030 [file mixoa_sm_r3_fbad030.pdf]

Feeding in mixoplankton enhances phototrophy increasing bloom-induced pH changes with ocean acidification

Kevin J Flynn and Aditee Mitra

SUPPLEMENTARY MATERIAL

Supplementary Methods

Protist model function description

In **Fig. 1** the salient differences between protist plankton physiologies are shown. The main physiological processes described in the model are shown in **Fig. 2**, with a schematic of the model showing the state variable connections given in **Fig. S1** for the CM variant.

The rationale for the model, and detailed descriptions are given in Flynn (2021), Mitra and Flynn (2021) and Flynn and Mitra (2023). Here an overview is given in the form of functional equations.

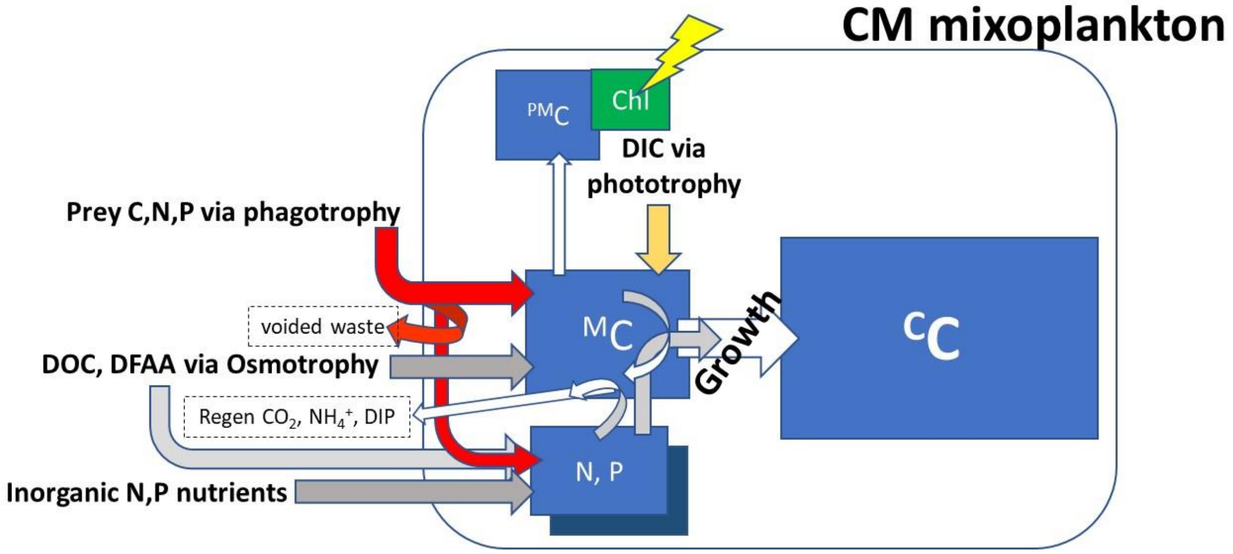

Fig. S1. Diagrammatic representation of the model, configured for a constitutive mixoplankton. Interconnectivity is shown between the state variables for structural C (^{C}C), metabolite C (^{M}C), N and P biomass, C-biomass allocated to the photosystem (chloroplast; ^{PM}C), and chlorophyll (Chl). This is shown specifically for a constitutive mixoplankton (CM). Forms of dissolved organic matter (DOM) are shown as dissolved organic C (DOC) and dissolved free amino acids (DFAA). Inorganic nutrients include dissolved inorganic P (DIP), ammonium and nitrate. Total cell-C, ^{T}C, = ^{C}C+^{M}C+^{PM}C. The C-status is related to ^{M}C:^{T}C, N-status to N:^{T}C and P-status to P:^{T}C. Pool ^{M}C equates to the 'metabolic pool' in **Fig. 2**.

Functional Equations

The following provides a functional-equation description of the model as text strings, with the form:

result = *f*{comma delimited list of factors involved in deriving the result}

Underlined terms in the equations are rates.

Terms in bold donate those that provide a positive interaction (i.e., the result increases when the term increases; these are usually enacted via a curvi-linear function); terms not in bold may involve negative or more complex interactions (such as bell-shaped for the prey allometry that affects capture).

The equations are provided working backwards from the emergent organism growth rate, with descriptions of the steps enabling that rate to be attained.

Total protist biomass C is given as, $^{T}C = {}^{M}C + {}^{PM}C + {}^{C}C$.

The model comprises the following state variables and associated flows (see also **Fig. S1**):

Core structure: prot^{C}C; mgC m^{-3}

d prot^{C}C/dt = {anabolism} – {catabolism when $^{M}C{:}^{T}C$ is critically low}

Metabolic pool of C: prot^{M}C ; mgC m^{-3}

d prot^{M}C/dt = {osmotrophy(C)} + {phagotrophy(C)} + {phototrophy} + {degradation of prot^{PM}C} – {synthesis of prot^{PM}C} – {anabolism} – {catabolism} – {NO3-assimilation(reductant)} – {DOM(C)-leak}

C in chloroplasts : prot^{PM}C; mgC m^{-3} (this sets the maximum rate of photosynthesis, Pmax)

d prot^{PM}C/dt = {synthesis of ^{PM}C} – {degradation of ^{PM}C}

Photopigments : protChl; mgChl m^{-3}

d protChl/dt = {synthesis of Chl} – {degradation of Chl}

Organism-P : protP ; mgP m^{-3}

d protP/dt = {osmotrophy(P)} + {phagotrophy(P)} + {DIP-assimilation} – {P-regeneration} – {DOM(P)-leak}

Organism-N : protN; mgN m^{-3}

d protN/dt = {osmotrophy(N)} + {phagotrophy(N)} = {NH4-assimilation} + {NO3-assimilation} – {N-regeneration} – {DOM(N)-leak}

Depending on the application, additional state variables are included:

- **protCells** (cells m^{-3}): cells, required for a dynamic description of cell-size with nutrient status, diel light cycle and temperature
- **protSi** (mgSi m^{-3}); organism-Si, required for diatoms
- **protANA** (mgNA m^{-3}); acquired nucleic acid material from phototrophic prey, required to support acquired phototrophy in plastidic specialist non-constitutive mixoplankton (pSNCM)

NOTE: In the functional equations below, for brevity, state variable names in the equations do not include the prefix 'prot'.

Growth and nutrient status

Ultimately growth is a function of the nutritional status of the organism (in terms of elements C, N and P) and the maximum growth rate potential. The latter varies with temperature, T, around the value of μ_{max} at a reference temperature, μ_{maxRT}.

growth = *f*{**C-status**, **N-status**, **P-status**, **μ_{max}**, losses}

μ_{max} = *f*{**μ_{maxRT}**, **T**}

The nutrient status defines the health of the organism in terms of C ($^{M}C{:}^{T}C$), N ($N{:}^{T}C$) and P ($P{:}^{T}C$), and is a function of various inputs and outputs. Inputs are associated with the use of dissolved organic substrates via osmotrophy, prey via phagotrophy, and also the use of inorganics via phototrophy. Losses occur through respiration and regeneration, and also through the leakage of metabolites as dissolved organic matter (DOM), some of which may be recovered via osmotrophy.

C-status = *f*{**osmotrophy**, **phagotrophy**, **phototrophy**, C-respiration, DOM-leak}

N-status = *f*{**osmotrophy**, **phagotrophy**, **phototrophy**, **DIN assimilation**, N-regeneration, DOM-leak}

P-status = *f*{**osmotrophy**, **phagotrophy**, **phototrophy**, **DIP assimilation**, P-regeneration, DOM-leak}

Losses = *f*{**C-respiration**, **N-regeneration**, **P-regeneration**, **DOM-leak**}

Growth is associated with catabolic (including basal) and anabolic respiration, part of which is associated with specific dynamic action (SDA) during prey digestion and assimilation. Anabolic respiration is affected by the flows of resources via the different trophic mechanisms. Nitrate assimilation incurs an additional cost for reduction of nitrate to nitrite to ammonium. There are also losses of C, N, P required to preserve organism stoichiometry within the bounds of acceptable C:N:P.

C-respiration = f{**μ_{max}**, **basal respiration**, prot-C:N, **C-assimilation**, **NO3-assimilation**}

C-assimilation = f{**osmotrophy**, **phagotrophy**, **phototrophy**}

N-regeneration = f{**C-respiration**, **prot-C:N**, **prey-C:N**, **digestion**, SDA}

P-regeneration = f{**C-respiration**, **prot-C:P**, **prey-C:P**, **digestion**, SDA}

DOM-leak is closely associated with osmotrophy (see further below).

Cell division occurs when the cell reaches a critical size (which varies with nutrient status and temperature affecting the growth rate), and typically occurs in phototrophs within a specific part of the diel light:dark (LD) cycle.

division = *f*{**size**, critical size, LD}

critical size = *f*{T, C-status, N-status, **P-status**, **growth**}

The size of the organism affects predation for phagotrophy, and whether it itself is likely to encounter its own predator.

Osmotrophy

Osmotrophy depends on the concentration of the substrate, [DOM], the C:N:P status of that material, and the uptake kinetics parameters of the maximum uptake rate ($^{DOM}V_{max}$) and the substrate affinity (i.e., $^{DOM}V_{max}/K_{DOM}$). The uptake kinetics depend on the nutrient status of the organism; cells that are nutrient-stressed have a higher uptake potential and a high affinity.

osmotrophy = *f*{**[DOM]**, DOM-C:N:P, $\mathbf{^{DOM}V_{max}}$, $\mathbf{^{DOM}V_{max}/K_{DOM}}$}

$^{DOM}V_{max}$ = *f*{C-status, N-status, $\mathbf{\mu_{max}}$}

$1/K_{DOM}$ = *f*{C-status, N-status}

Against the gains from osmotrophy there are losses with the leakage of DOM. At especially high growth rates, which require a high nutrient status and hence a cell that is replete with the internal metabolite pool containing mM concentrations, DOM inevitably leaks. Osmotrophy may recover some of that leakage. The net leakage of N-containing DOM (as amino acids) is most significant during N-replete growth conditions, while leakage of DOC (sugars) occurs especially with high rates of phototrophy, including when N becomes exhausted and the cell has yet to down-regulate photosynthesis.

DOM-leak = f{C-status, N-status, **osmotrophy**, **phagotrophy**, **phototrophy**, $\mathbf{\mu_{max}}$}

Phagotrophy and voiding of waste

Phagotrophy brings in resources from the assimilation of prey biomass; note the plural in prey-assimilations in the equations. Prey need to be encountered (which depends on the sizes of the predator organism and of the prey, their respective motilities and turbulence), captured (which, like predator motility, varies with satiation, and also with the 'taste' of the prey as affected by its stoichiometric quality), and then ingested. These processes are prey-species specific; the collective biomass from many ingestions, perhaps of different prey organisms, is then digested. During digestion a fraction of the ingested prey is subjected to voiding (depending on the assimilation efficiency, AE, predator satiation and the food quality), and another fraction is lost associated with specific dynamic action (SDA) as the prey biomass is subjected to catabolic and then anabolic processes. The internal recycling of regenerated inorganic nutrients is a critical step in mixoplankton (**Fig. 2**; see *Inorganic nutrient assimilations*, below).

phagotrophy = *f*{**prey-assimilations**}

prey-assimilation = *f*{**digestion**, SDA}

digestion = *f*{**ingestion**, voiding, **prey C:N:P**, μ_{max}}

ingestion = *f*{**capture**, μ_{max}}

capture = *f*{C-status, N-status, P-status, **prey quality**, prey allometry}

encounter = *f*{**[prey]**, allometry, **motility**, **prey motility**, **turbulence**}

motility = *f*{C-status, N-status, P-status}

voiding = *f*{**ingestion**, **C-status**, **N-status**, **P-status**, prey quality, AE}

Phototrophy

Photosynthesis depends on light, the availability of dissolved organic C (DIC, especially as CO_2 and HCO_3^-), photopigment content (Chl:C), the value of alpha governing the initial slope of the light-photosynthesis curve, and the maximum rate of C-fixation (P_{max}). The value of P_{max} is set by the size of ^{PM}C:^{T}C. For organisms with a constitutive ability to photosynthesise, both Chl:C and P_{max} are modulated by the demand for C and energy, reflected by the organisms' nutritional status and growth rate potential. For non-constitutive mixotrophs (NCM), phototrophy is acquired from captured phototrophic prey. Light is a function of the photon flux density at the water surface and of attenuation within the water (which varies with the biomass of the pigmented organisms). Light also varies over the diel light:dark cycle; this imparts a diel cycle on phototrophy that then feeds through to affect osmotrophy and phagotrophy via feedback processes.

phototrophy = *f*{light, **[DIC]**, **Chl:C**, **alpha**, P_{max}}

Chl:C = *f*{C-status, **N-status**, μ_{max} (for NCM, prey-Chl:C, capture)}

P_{max} = *f*{C-status, **N-status**, P-status, μ_{max} (for NCM, prey-P_{max}, capture)}

Inorganic nutrient assimilations

Inorganic nutrients are sourced both internally, as regenerative products of prey assimilation, and externally; use of the former takes priority and will be affected by prey C:N:P. The use of external nutrients depends on the substrate concentrations ([DIP], [NH4], [NO3]) and the respective uptake kinetics (uptake-Vmax, affinity).

The latter vary with the nutritional state of the organism, with uptake potential enhanced when nutrient-stressed and, at the extreme, shut down if nutrient-replete (i.e. uptake-Vmax tends to zero at elevated nutrient status).

DIP assimilation = f{prey-assimilation, prey C:N:P, SDA, **[DIP]**, **$^{DIP}V_{max}$**, **$^{DIP}V_{max}/K_{DIP}$**}

$^{DIP}V_{max}$ = f{P-status, **μ_{max}**}

$1/K_{DIP}$ = f{P-status}

The uptake of DIN is affected also by the P-status of the organism. The uptake kinetics for ammonium (NH4) provide for development of an enhanced uptake capability over that for nitrate (NO3), with that development also commencing at a higher N-status. The latter results in ammonium being taken up 'in preference' to nitrate. There is no 'inhibition' term controlling NO3-assimilation by [NH4]; if the supply of ammonium from internal recycling plus external sources cannot meet the demand, then the ability to use nitrate is de-repressed.

DIN assimilation = f{prey-assimilation, prey C:N:P, SDA, **NH4-assimilation**, **NO3-assimilation**}

NH4-assimilation = f{**[NH4]**, **$^{NH4}V_{max}$**, **$^{NH4}V_{max}/K_{NH4}$**}

NO3-assimilation = f{**[NO3]**, **$^{NO3}V_{max}$**, **$^{NO3}V_{max}/K_{NO3}$** }

$^{NH4}V_{max}$ = f{N-status, P-status, **μ_{max}**}

$1/K_{NH4}$ = f{N-status}

$^{NO3}V_{max}$ = f{N-status, P-status, **μ_{max}**}

$1/K_{NO3}$ = f{N-status}

Controlling physiological processes

The model uses the normalised value of the nutrient quotas (i.e., 0 at minimum quota, 1 at optimal quota) for C, N and P (see **Fig. S2**) to control physiological processes. In essence these controls replicate the biochemical events of (de)repression and, consistent with allosteric controls, exploit sigmoidal curve functions. For example, the N:C quota value is used to controls the use of ammonium and nitrate (see Flynn 2001).

The value of RelMC (defined as $^{M}C:^{T}C$; see **Fig. S1**) was used to modulate the (de)repression of phototrophy and phagotrophy in the work described here.

The curve form for CCu is:

CCu=(1+CCuK^CCuH)*RelMC^CCuH/(RelMC^CCuH+CCuK^CCuH)

For the curve shown in **Fig. S3**, CCuH=4, CCuK=0.2. The model is not sensitive to the value of these parameters.

The generic curve form for the resource acquisition controls is:

Con=(1+K^H)*(1-RelMC)^H/((1-RelMC)^H+K^H))

For the PS and Pred curves shown in **Fig.S2**, H=8 and K=0.4. For the more repressed curves ('phag>phot', or 'phot>phag'), H=8 and K=0.8.

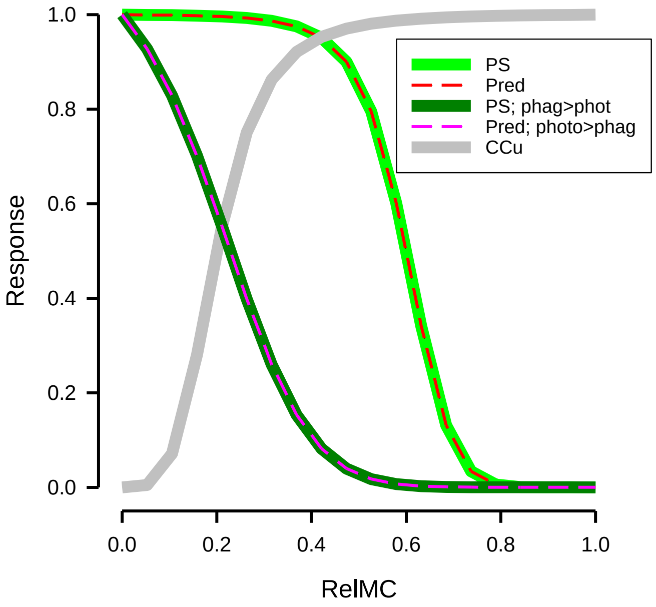

Fig. S2. Control of growth, resources acquisition via phototrophy and phagotrophy through reference to the value of RelMC. The relative availability of C for growth is defined by CCu, increasing as the relative size of the metabolite pool (RelMC) increases. Curves describing the (de)repression of phototrophy and phagotrophy are shown for when they are similar (PS and Pred respectively), and also for when either of these processes is de-repressed when the cell is of a lower C-status (i.e. RelMC is lower). Shifting these curves provides a mechanism to alter the balance of phagotrophy vs phototrophy ('phag>phot', or 'phot>phag'). Note that the cross-over point between CCu and the resource acquisition trophic control curves affects the potential maximum growth rate attainable when solely exploiting that trophic mode. For example, when phagotrophy provides a backup mechanism for when phototrophy cannot supply sufficient C to maintain a high enough RelMC ('Pred; photo>phag'), this aligns with a growth rate maximum exploiting phagotrophy of ca. 50% (Response ≈0.5).

For phagotrophy (which brings in a complete package of C,N,P) the above described control dominates regulation of the activity. However, phototrophy is also controlled by the acquisition of N (as ammonium and nitrate) and P (as phosphate); those interactions are modulated by N:C and P:C respectively. Phototrophy is further affected by synthesis and operation of the photosystems, which are modulated via RelMC and N:C.

Table S1 Key parameter values for model configurations. As far as possible, consistent with the literature, parameter values were kept the same across protist functional types.

Explanation	Unit	Common	zoo	GNCM	pSNCM	CM	phyto
photon flux density	µmol m^{-2} s^{-1}	500					
light:dark ratio	-	0.7:0.3					
salinity	-	35					
temperature	°C	15					
initial NH4+	mgN m^{-3}	140					
initial NO3-	mgN m^{-3}	140					
initial DIP	mgP m^{-3}	38.75					
initial prey biomass	mgC m^{-3}	1647					
prey ESD	µm	5					
prey N:C stoichiometry	mass ratio	0.17					
prey P:C stoichiometry	mass ratio	0.024					
prey Chl:C stoichiometry	mass ratio	0.06					
prey alpha	*	7E-06					
prey photosystemC:totalC	mass ratio	0.05					
initial protist biomass	mgC m^{-3}	5					
minimum cell size	pgC $cell^{-1}$	400					
maximum cell size	pgC $cell^{-1}$	1000					
emergent average ESD	µm	20					
protist maximum growth rate	d-1	0.7					
maximum protist N:C	mass ratio	0.2					
optimum protist N:C	mass ratio	0.15					
minimum protist N:C	mass ratio		0.1	0.1	0.07	0.05	0.05
NC quota curve constant	-	10					
maximum protist P:C	mass ratio	0.05					
optimum protist P:C	mass ratio	0.024					
minimum protist P:C	mass ratio	0.005					
PC quota curve constant	-	0.1					
proportion of encountered prey captured	-		0.2	0.2	0.2	0.2	
minimum assimilation efficiency	-		0.3	0.3	0.3	0.3	
maximum assimilation efficiency	-		0.6	0.6	0.6	0.6	
maximum protist Chl:C	mass ratio			0.03	0.03	0.03	0.03
protist alpha	*			7E-06	7E-06	7E-06	7E-06
maximum protist PSmax:Umax	-			1	4	4	4

The unit for alpha is (m^2g^{-1} chl.a)(gC µmol^{-1} photon)

Modelling the carbonate chemistry system, alkalinity and pH

The following describes the ocean acidification sub-model, including the carbonate chemistry system and changes in pH and alkalinity with biological activity. This is the sub-model used by Flynn et al. (2012, 2015, 2016).

The model was operated with an Euler integration routine (step size 0.015625 d). As described here, it was configured to enable an exploration of the effects of changing different abiotic parameters and also of the balance of phototrophic vs heterotrophic events.

The model includes traps to halt the simulation if certain conditions are met, notably if a state variable goes negative, or if the operator has requested that the model pauses at regular times to enable changes in operational parameters. Changes in nutrients affect alkalinity and the size of the carbonate system which provides the greater proportion of seawater pH buffering. Here those biotic-linked changes are controlled by the constant deltaC and C:N:P ratios etc.

Calculations of the sea water chemistry operate using units of μmol kg^{-1}, requiring various transforms and corrections with salinity and temperature from the units used to describe the biological functionality. Gas exchange of CO_2 (if enabled) moves DIC in and out of the system at the water surface as functions of wind speed, atmospheric pCO_2, salinity and temperature. Calculation of the different components of the carbonate system responsible for most of the seawater buffering are also related to salinity and temperature as well as the current acidity. Calculation of omega (dissolution of) calcite, are included here for completeness.

Changes in total alkalinity occur with physiology. There are decreases (i.e. TA_out is +ve) with ammonium use and with calcification, and increases (i.e. TA_out is -ve) with nitrate and phosphate use. TA_out is effectively TA uptake into organisms. For this implementation, rates of biological action are just set using the constant deltaC. The calculation of acidity and total alkalinity is an iterative process as changes in each affect the other via dissociations of the key ionic compounds.

Table S2 Constants for the carbonate chemistry, alkalinity and pH module.

Constant	Value	Unit	Description
Borate	415	mmol m^{-3}	total Boron, equiv to 0.0004151 mol/Kg = approx 0.4151 mol/m3
c	4.83E-04		constant for calculation of water density based on T and S
CaC	0*40.08/12	gCa gC^{-1}	PICa:POC assume PIC:POC =1, and CaCO3 which has a ratio of 40.08:12
deltaC	0	mgC m^{-3} d^{-1}	rate of C-fixation (+ve) or respiration (-ve)
deltaCa	0	mgCa m^{-3} d^{-1}	rate of Ca incorporation
deltaDIP	0	mgP m^{-3} d^{-1}	change in DIP; to be used only when deltaC=0 to check impact of just this contribution
deltaNH4	0	mgN m^{-3} d^{-1}	change in NH4; to be used only when deltaC=0 to check impact of just this contribution
deltaNO3	0	mgN m^{-3} d^{-1}	change in NO3; to be used only when deltaC=0 to check impact of just this contribution
deltaSi	0	mgSi m^{-3} d^{-1}	change in Si; to be used only when deltaC=0 to check impact of just this contribution
dil	0	d^{-1}	dilution rate (this equates to total mixing rate in an oceanographic setting)
f	0		f-ratio (1 if all NO3, 0 if NH4); defaults to 0 if deltaC is negative for regeneration
initCa	10000*40.078	mgCa m^{-3}	Ca concentration; assume typical is ca. 10282.1 umol/Kg, ca. 10000uM, with at.wt 40.078
initDIC	24000	mgC m^{-3}	DIC
initDIP	32*5	mgP m^{-3}	DIP nutrient concentration
initNH4	14*50	mgN m^{-3}	NH4 nutrient concentration
initNO3	14*50	mgN m^{-3}	NO3 nutrient concentration
initpH	8	pH	initial pH; note that altering this also alters the initial TA value automatically
initSi	28*50	mgSi m^{-3}	Si nutrient concentration
MLD	0.1	m	mixed layer depth
NC	0.176100629	gN gC^{-1}	N:C in organism
pauseT	20	d	simulation pause frequency
PC	0.024371069	gP gC^{-1}	P:C in organism
PCO2A	400	atm	atmospheric pCO_2
S	35		salinity
SiC	0*28/14/7	gSi gC^{-1}	ratio of Si:C in diatom typical Si:N is 1, and N:C@1/7
Sw_CO2dif	1		switch to allow gas exchange; 0 if no exchange; 1 if allowed
T	10	°C	temperature
Wnd	0.1*100	m s^{-1}	wind speed

Table S3 State variables, including their initial values and flows, for the carbonate chemistry, alkalinity and pH module.

State Variable	Initial Value	Flow	Unit	Definition
Ca	initCa	-Caout	mgCa m^{-3}	Ca concentration
DIC	initDIC	+CO2ex<br>-DICout	mgC m^{-3}	DIC total of all forms
DIP	initDIP	-DIPio	mgP m^{-3}	DIP nutrient concentration
H	10^(-initpH)	+H_mix<br>+H_up<br>-H_down	mol kg^{-1}	Protons in bulk water
H_aby	H		mol kg^{-1}	Protons in abyss
NH4	initNH4	-NH4io	mgN m^{-3}	NH4 nutrient concentration (technically, NH_4^+ + NH_3)
NO3	initNO3	-NO3o	mgN m^{-3}	NO3 nutrient concentration
Si	initSi	-Sio	mgSi m^{-3}	Si nutrient concentration
TA	TAc	+TA_mix<br>-netTA_out	µmol kg^{-1}	bulk water Total Alkalinity
TA_aby	TAc		µmol kg^{-1}	value of TA in abys

Table S4 Variables for the carbonate chemistry, alkalinity and pH module.

Variable	Value	Unit	Definition
a_1	824.49300e-3 - 4.0899e-3*T + 76.4380e-6*T^2 - 824.6700e-9*T^3 + 5.3875e-9*T^4		constant for calculation of water density based on T and S
b - 2	-0.00572466 + 0.00010227*T - 0.0000016546*T^2		constant for calculation of water density based on T and S
BOH4	Borate/(1+H/Kb)	µmol kg^{-1}	current B(OH)4 concentration
Cam	uMCa/dcf	µmol kg^{-1}	calcite converted to mass units
Caout	IF(Ca>0,Caup)	mgCa m^{-3} d^{-1}	removal of Ca
Caup	IF(deltaC>0,deltaC*CaC)+IF(deltaC=0 AND deltaCa>0,deltaCa)	mgCa m^{-3} d^{-1}	removal of Ca
CO2_Flux	Fwind * HENRY * (PCO2A - pCO2w)/1e6 * dcf	mmol m^{-2} d^{-1}	flux of CO2 in/out of water surface (NOTE that the equation works on atm, not uatm, hence the 1e6)
CO2ex	Sw_CO2dif*12*CO2_Flux/MLD	mgC m^{-3} d^{-1}	change in DIC with CO2 exchange
CO3m	(k1*k2/(H^2+H*k1+k1*k2))*DICm	µmol kg^{-1}	current CO_3^{--} concentration
dcf	(999.842594 + 67.939520e-3*T- 9.095290e-3*T^2 + 100.168500e-6*T^3 - 1.120083e-6*T^4 + 6.536332e-9*T^5 + a_1*S + 'b - 2'*S^1.5 + c*S^2)/1.0e3		correction factor for water density at given T and S; to convert µmol/Kg to µmol/L multiply by dcf
DICm	uMDIC/dcf	µmol kg^{-1}	DIC converted to mass units
DICout	IF (DIC>500,deltaC+Caup*12/40.078)	mgC m^{-3} d^{-1}	removal of DIC into biomass, plus (if applicable) into CaCO3
DIPio	IF(DIP>0,DIPup-Preg)	mgP m^{-3} d^{-1}	DIP in/out
DIPm	uMPO4/dcf	µmol kg^{-1}	umole Kg-1 uM DIP converted to mass units
DIPup	IF(deltaC>0,deltaC*PC)+IF(deltaC=0 AND deltaDIP>0,deltaDIP)	mgC m^{-3} d^{-1}	increase in C-biomass
Fatmk	EXP(218.2968*(100/TK) - 162.8301 + 90.9241 * LN(TK/100) - 1.47696 * (TK/100)^2 + S* (0.025695 - 0.025225 * (TK/100) + 0.0049867 * (TK/100)^2))		intermediate in calculation of pCO2w
fCO2	H2CO3m/k0	ppm	fugicity of CO_2
Fwind	24/100 * (0.222 * Wnd^2 + 0.333 * Wnd)*(sc/660)^-0.5	m d^{-1}	Fwind
H2CO3m	(H^2/(H^2+H*k1+k1*k2))*DICm	µmol kg^{-1}	$H_2CO_3^-$
H2PO4	(DIPm *K1P*H^2)/(H^3 + K1P*H^2 + K1P*K2P*H + K1P*K2P*K3P)	µmol kg^{-1}	H_2PO_4
H3PO4	(DIPm*H^3)/(H^3 + K1P*H^2 + K1P*K2P*H +K1P*K2P*K3P)	µmol kg^{-1}	H_3PO_4
H9	H*1e9	nmol kg^{-1}	proton concentration * 1e9
H9L	H9*dcf	nmol L^{-1}	proton concentration L^{-1}
H_down	IF((TAc<TA), 1, 0)*1e-6*(1-TAc/TA)	mol kg^{-1} d^{-1}	correction of H to match TAc and TA; TAc too low so decrease H (increase pH)
H_mix	dil*(H_aby-H)	mol kg^{-1} d^{-1}	dilution (mixing) of protons in/out
H_up	IF((TAc>TA), 1, 0)*1e-6*(1-TA/TAc)	mol kg^{-1} d^{-1}	correction of H to match TAc and TA; TAc too high so increase H (decrease pH)
HCO3m	((H*k1)/(H^2+H*k1+k1*k2))*DICm	µmol kg^{-1}	HCO_3^- concentration
HENRY	EXP(Hint3)*1000*1000	mmol m^{-3} atm^{-1}	Henry constant for CO2 , expected value of around 34x10^-2 mol/L/atm. EXP(Hint3) gives values as mol/L/atm
Hint1	(-574.7012600e0)+(21.541520e3)/TK+(-147.75900e-6)*TK^2+(89.8920e0)*LN(TK)		intermediate for HENRY; Ln(K0'(T))
Hint2	(29.9410e-3)+(-274.5500e-6)*TK+(534.0700e-9)*TK^2		intermediate for HENRY; f(T)
Hint3	Hint1+Hint2*S		intermediate for HENRY; Ln(Ko'(T,S))
HPO4	(DIPm *K1P*K2P*H)/(H^3 + K1P*H^2 + K1P*K2P*H + K1P*K2P*K3P)	µmol kg^{-1}	HPO_4^{--}
k0	EXP(93.4517/(TK/100.0) - 60.2409 + 23.3585 * LN(TK/100.0)+S * (.023517 - 0.023656 * (TK/100.0) + 0.0047036 * (TK/100.0)^2))		dissociation constant for H_2CO_3 taking into account TK and S
k1	10^(-1*(3670.7/TK - 62.008 + 9.7944*LN(TK) -0.0118 * S + 0.000116*S^2))		dissociation constant for HCO_3 taking into account TK and S
K1P	EXP(-4576.752/TK + 115.525 - 18.453 * LN(TK) + (-106.736/TK + 0.69171)*S^0.5 + (-0.65643/TK - 0.01844)*S)		dissociation constant for DIP taking into account TK and S
k2	10^(-1*(1394.7/TK + 4.777 - 0.0184*S + 0.000118*S^2))		dissociation constant for CO_3 taking into account TK and S
K2P	EXP(-8814.715/TK + 172.0883 - 27.927 * LN(TK) + (-160.34/TK + 1.3566)*S^0.5 + (0.37335/TK - 0.05778)*S)		dissociation constant for DIP taking into account TK and S
K3P	EXP(-3070.75/TK - 18.141 + (17.27039/TK + 2.81197)*S^0.5 + (-44.99486/TK - 0.09984)*S)		dissociation constant for DIP taking into account TK and S

Table S4 continued

Variable	Value	Unit	Definition
Kb	EXP((-8966.90 - 2890.53*S^0.5 - 77.942*S + 1.728*S^1.5 - 0.0996*S^2)/TK + (148.0248 + 137.1942*S^0.5 + 1.62142*S) + (-24.4344 - 25.085*S^0.5 - 0.2474*S) * LN(TK) + 0.053105*S^0.5 *TK)		dissociation constant for borate taking into account TK and S
Kcal	10^(-171.9065 - 0.077993*TK + 2839.319/TK + 71.595*LOG(TK) + (-0.77712 + 0.0028426*TK + 178.34/TK)*S^0.5 - 0.07711*S + 0.0041249*S^1.5)		dissociation constant for calcite formation taking into account TK and S
Knh3	EXP(-6285.33/TK + 0.0001635*TK - 0.25444 + (0.46532 - 123.7184/TK)*S^0.5 + (-0.01992 + 3.17556/TK)*S)		dissociation constant for NH3
KSiOOH3	EXP(-8904.2/TK + 117.385 - 19.334*LN(TK) + (-458.79/TK + 3.5913)*(0.02*S)^0.5 + (188.74/TK - 1.5998)*(0.02*S) + (-12.1652/TK + 0.07871)*(0.02*S)^2 + LN(1-0.001005*S))		dissociation constant for SiOOH3
Kw	EXP(-13847.26/TK+148.9652-23.6521*LN(TK)+(118.67/TK-5.977+1.0495*LN(TK))*S^0.5 - 0.01615*S)		dissociation constant for H2O taking into account TK and S
maxCO3	IF(STOPIF(CO3m/DICm>0.95), 1, 0)		trap if CO3-- becomes too high
maxpH	IF(STOPIF(H<1e-12), 1, 0)		trap if pH becomes too high
netTA_out	(TAout-TAin)/dcf	µmol kg^{-1} d^{-1}	change in TA with biological action
NH3	NH4m / (1 + H/Knh3)	µmol kg^{-1}	NH3
NH4io	IF(NH4>0,NH4up-Nreg)	mgN m^{-3} d^{-1}	NH4 in/out
NH4m	uMNH4/dcf	µmol kg^{-1}	NH4 converted to mass units (this is actually NH4+NH3)
NH4up	IF(deltaC>0,deltaC*NC*(1-f))+IF(deltaC=0 AND deltaNH4>0,deltaNH4)	mgN m^{-3} d^{-1}	uptake of NH4 into algal biomass
NO3o	IF(NO3>0,NO3up)	mgN m^{-3} d^{-1}	NO3 out
NO3up	IF(deltaC>0,deltaC*NC*f)+IF(deltaC=0 AND deltaNO3>0,deltaNO3)	mgN m^{-3} d^{-1}	uptake of NO3 into protist biomass
Nreg	IF(deltaC<0,-1*deltaC*NC)+IF(deltaC=0 AND deltaNH4<0,-1*deltaNH4)	mgN m^{-3} d^{-1}	NH4 release by regeneration
OH	Kw/H*1e6	µmol kg^{-1}	OH concentration
Omega_cal	(Cam/1e6)*(CO3m/1e6)/Kcal		saturation value for calcite - note this requires mol / kg values hence 1e6 corrections. A value of 1 is equilibrium; >1 indicates supersaturation
PauseCon	PAUSEIF(TIME=pauseT)		pause control of simulation; pauses simulation every multiple of simulation TIME defined by pause T
pCO2w	H2CO3m/Fatmk	atm	water pCO_2
pH	-1*(LOG(H))	pH	pH
PO4	(DIPm *K1P*K2P*K3P)/(H^3 + K1P*H^2 + K1P*K2P*H + K1P*K2P*K3P)	µmol kg^{-1}	PO4
Preg	IF(deltaC<0,-1*deltaC*PC)+IF(deltaC=0 AND deltaDIP<0,-1*deltaDIP)	gP g^{-1}C d^{-1}	regeneration of P; if P:C exceeds PCmax then this excess is voided
sc	2073.1-125.62*T + 3.6276*T^2 - 0.0432190*T^3		Schmidt number
Sim	uMSi/dcf	µmol kg^{-1}	Si converted to mass units
simCon	STOPIF(Ca*DIC*DIP*NH4*NO3*Si<=0)		trap to halt the simulation if a substrate is exhausted
Sio	IF(Si>0,Siup)	mgSi m^{-3} d^{-1}	Si out
SiOOH3	Sim/(1+H/KSiOOH3)	µmol kg^{-1}	SiOOH3
Siup	IF(deltaC>0,deltaC*SiC)+IF(deltaC=0 AND deltaSi>0,deltaSi)	mgSi m^{-3} d^{-1}	removal of Si
TA_mix	dil*(TA_aby-TA)	µmol kg^{-1} d^{-1}	TA exchange in/out
TAc	HCO3m+2*CO3m+BOH4+OH+HPO4+2*PO4+SiOOH3+NH3-(H*1e6)-H3PO4	µmol kg^{-1}	total alkalinity with current operational H+ (umol / kg) {note that H+ is as mol / Kg, hence *1e6}
TAin	(NO3up+Nreg)/14+DIPup/31	mmol m^{-3} d^{-1}	addition of TA taking into account the molecular wt of the ions and their (negative) charges
TAout	NH4up/14+Preg/30.97+2*Caup/40.078	mmol m^{-3} d^{-1}	removal of TA taking into account the molecular wt of the ions and their (positive) charges
TK	T+273.15	K	absolute temperature
uMCa	Ca/40.078	µM	bulk Ca
uMDIC	DIC/12	µM	bulk DIC
uMNH4	NH4/14.0	µM	bulk NH4
uMNO3	NO3/14	µM	bulk NO3
uMPO4	DIP/31.0	µM	bulk DIP
uMSi	Si/28	µM	bulk Si

The consequences of phototrophy vs heterotrophy on seawater acidity starting at equilibria with pCO_2 = 300 atm or 600 atm, are demonstration in **Fig. S3**. This shows the consequence of phototrophy or heterotrophy for seawater acidity (i.e., for changes in [H^+]) and provides an insight of the changes in acidity that develop during protist plankton growth especially as the different protist functional groups release or take up different inorganic nutrients that affect alkalinity and thence pH.

Positive values of δ (i.e., an increase in δ C for biomass in **Fig. S3**) shows the impact of phototrophy with C-fixation removing CO_2 from the seawater with or without concurrent assimilation of dissolved inorganic P (DIP) and dissolved inorganic N (DIN, as ammonium or as nitrate). Differences between these plots show how the nutrient assimilations affect alkalinity and thus further modify the impact of the C-fixation upon acidity. Thus, the use of ammonium (the removal of which decreases alkalinity) increases acidity and partially counters the basification caused by CO_2 removal with C-fixation. Phototrophy using nitrate increases basification. Heterotrophic processes, as occur with phagotrophy, are associated with respiration (i.e., a negative δ C for biomass in **Fig. S3)**; this adds CO_2 to the seawater causing acidification. With that respiration there is also a regeneration of DIP and DIN (as ammonium only). The addition of ammonium raises alkalinity, partly compensating for the acidification caused by CO_2 release.

Changes in [H^+] in the 600 atm pCO_2 scenario, from an initial value almost double that in the 300 atm pCO_2 scenario, are greater because of the lower pH buffering capacity at the initial pCO_2 equilibrium state.

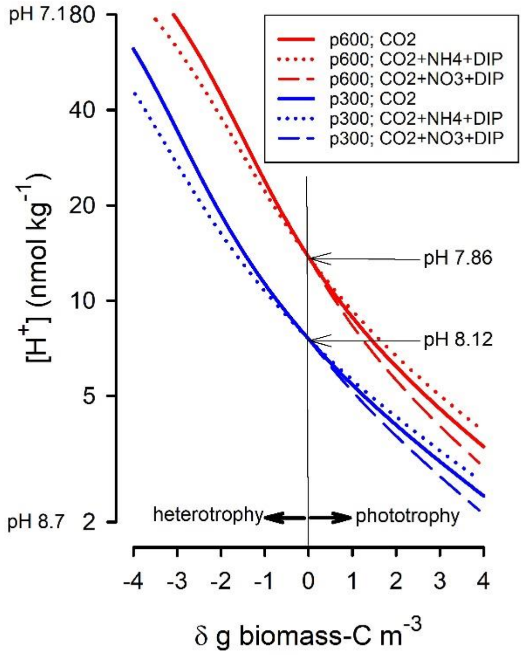

Fig. S3 Changes in [H^+] with phototrophy and heterotrophy. Decreases in [H^+] occur during C-fixation, associated with the production (δC >0) of organics and the concurrent inorganic nutrient assimilation. Increases in [H^+] occur during heterotrophy, associated with the loss (δ gC <0) of organics, and hence regeneration of CO_2 and inorganic nutrients. The equilibrium state (δ gC =0), in water of salinity 35 and at 15°C, was with pCO_2 of 300 atm (p300, blue lines) or 600 atm (p600, red lines), but thereafter excludes air-sea gas exchange. The different line types show changes due to just the assimilation or regeneration of CO_2 (solid lines), or additionally coupled with Redfield C:N:P assimilation or regenerations of phosphate and dissolved inorganic N. The N was either considered as ammonium ('NH4'; dotted lines), or for assimilation only as nitrate ('NO3'; dashed lines). For reference, the pH values are indicated at the equilibrium states and the minimum and maximum [H^+] values on the y-axis. Note the y-axis is a log scale.

Supplementary Results

Supplementary Tables

Table S5 Final protist biomass (ProtC mgC m^{-3}), DOC (mgC m^{-3}), VOC (mgC m^{-3}), $[H^+]$ (nmol kg^{-1}) and pH from the simulations shown in the indicated Figures. Note that DOC in GNCM (**Fig. 3**) increased during the early part of the simulation but was then taken up by the GNCM bringing DOC down to near zero. See Figure legends for further details.

Protist	Conditions	Fig.	Equilibrium (start) pCO_2	final ProtC	final DOC	final VOC	final $[H^+]$	final pH
Protist-zoo-plankton	default	3	300	425	0	659	9.3	8.0
			600				18.1	7.7
GNCM	default	3	300	1422	0	726	6.5	8.2
			600				11.3	7.9
pSNCM	default	3	300	4876	2392	666	1.4	8.9
			600				1.9	8.7
CM	default	3	300	6742	3576	676	0.6	9.2
			600				0.9	9.1
Phyto-plankton	default	3	300	4318	1377	0	1.6	8.8
			600				2.2	8.7
CM photo>phago	default	5	300	5756	1660	1010	1.2	8.9
			600				1.7	8.8
CM phago>photo	default	5	300	6677	893	661	1.3	8.9
			600				1.8	8.7
CM	low PFD	5	300	7333	3344	663	0.5	9.3
			600				0.8	9.1
CM	prey quantity (2x prey)	5	300	8572	2087	1419	0.7	9.2
			600				1.0	8.9
CM	prey quantity & quantity (2x prey; 0.5x N:C and P:C)	5	300	6433	3165	2451	0.7	9.2
			600				1.0	8.9

Supplementary Figures

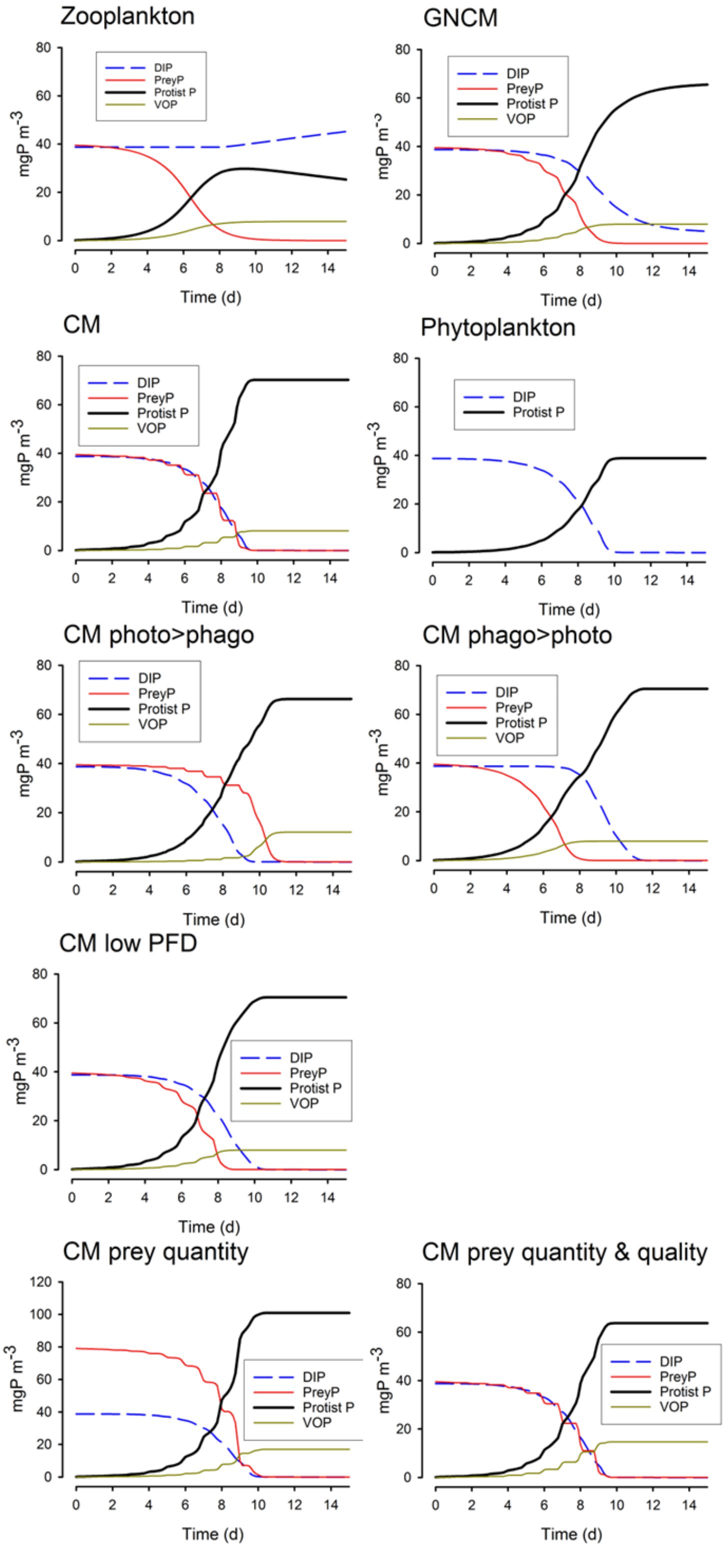

Fig. S4 The P dynamics for the simulations shown in **Figs. 3 & 5**. See legends to that figure for further details. Note the y-axis scale for 'CM prey quantity' is not the same as the scale for all the other plots.

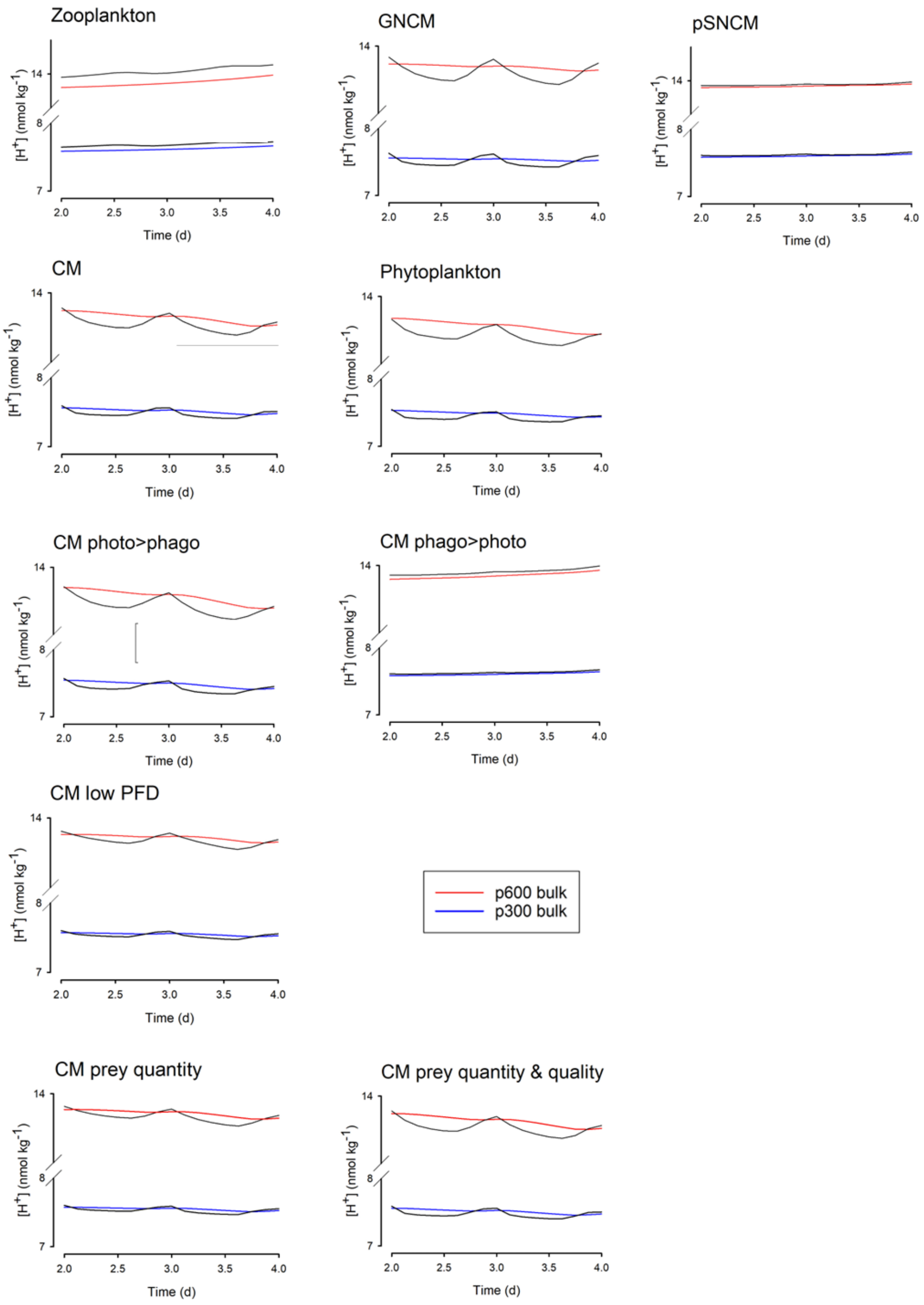

Fig. S5 Detail of near-cell (proximal) acidity for the early part of the simulations shown in Figs. 3, 4 and 6. See legends to those figures for further details. The black lines shows the proximal (near cell) acidity while the coloured lines show the bulk values. Simulations were run in closed systems originally equilibrated to pCO_2 of 300 (blue) or 600 atm (red). During each day, the first 0.7d was illuminated, hence the sinusoidal form of many of the proximal values. The two parts of the split y-axis for acidity are of equal value (i.e., 1 nmol kg^{-1}). Acidity increases with net heterotrophy, and decreases with net phototrophy. The greater difference between proximal and bulk acidities for the p600 series vs the p300 series is because of the lower buffering capacity in the seawater for the former scenario; the biological events were unaffected by acidity in these simulations (see also **Fig. S3**).

Supplementary Discussion Calculations

Balance of Phototrophic vs Phagotrophic CO_2 Dynamics

The following describes an estimation of the balance point between phagotrophy and phototrophy to achieve a net zero CO_2 exchange in a mixoplankton.

We assume the following tabulated constants. Dl – dimensionless

Parameter	Value	Unit	Description
Umax	0.693	gC gC^{-1} d^{-1}	Target growth rate
CR	0.05	Dl	Catabolic respiration (including basal respiration)
AE	0.8	Dl	Assimilation efficiency
SDA	0.3	Dl	Specific dynamic action
DOCfrac	0.1	Dl	Fraction of photosynthetic C-fixation lost as DOC
AR	1.5	gC gN^{-1}	Anabolic respiration
NC	0.15	gN gC^{-1}	Cellular N:C

The catabolic respiration (Cresp; gC gC^{-1} d^{-1}), is:

Cresp = Umax*CR

The assimilation rate from phagotrophy (Ass), where Ing is the ingestion rate (gC gC^{-1} d^{-1}), is given by:

Ass = Ing*AE*(1-SDA)-Cresp

The total respiration rate attributed to phagotrophy (PhagR; gC gC^{-1} d^{-1}) is then:

PhagR = Ing*AE*SDA+Cresp

The balance of C from phototrophy (PhotC; gC gC^{-1} d^{-1}) required to support Umax is:

PhotC = Umax-Ass

This C input from phototrophy is associated with the assimilation of ammonium described by PhotC*NC (gN gC^{-1} d^{-1}), which costs the amount of C described by AR. The anabolic respiration rate with phototrophy (PhotR; gC gC^{-1} d^{-1}) is:

PhotR = PhotC*NC*AR

The total C fixed and retained by the cell is PhotC+PhotR. However, we then need to add the C that is fixed but leaks out as DOC. The total C-fixation rate (GrossPS; gC gC^{-1} d^{-1}) is thus:

GrossPS = (PhotC+PhotR)*(1+DOCfrac)

The net CO_2 fixation with phototrophy (NetPS) is:

NetPS = GrossPS – PhotR

The balance of zero net CO_2 exchange is given when respiration from phagotrophy balances the net photosynthetic rate, i.e., when PhagR = NetPS.

With the above tabulated values, this balance is achieved with the input and output values given in the table below.

**Parameter**	**Value**	**Description** (all rates as gC gC^{-1} d^{-1})
Ing	0.9004	Ingestion rate (input value)
Cresp	0.03465	Catabolic respiration rate
Ass	0.46957	Assimilation rate of prey C
PhagR	0.25075	Total respiration rate not linked to phototrophy
PhotC	0.22343	CO_2 assimilation rate needed to attain Umax
PhotR	0.05027	Anabolic respiration rate with phototrophy
GrossPS	0.30107	Gross photosynthesis rate including DOC release
NetPS	0.25080	Net photosynthesis rate
PhagR-NetPS	0	Balance

From this, the ratio of total C inputs from phagotrophy to phototrophy is (Ing:GrossPS = 0.9:0.3 =) 3.

Using a value of DOCfrac=0, this ratio is 2.91

Supplementary References

Flynn, K. J. (2021). *Enhancing Microalgal Production - constructing decision support tools using system dynamics modelling.* Zenodo. http://doi.org/10.5281/zenodo.5036605

Flynn, K. J. and Mitra, A. (2023). *DRAMA - a cybernetic approach for Plankton Digital Twins.* Zenodo. https://doi.org/10.5281/zenodo.7848329

Mitra, A., Flynn, K. J., Konstantinos, A., Joost, M., Ferreira Guilherme, D. and Calbet, A. (2021) *Novel Approaches For Investigating Marine Planktonic Mixotrophy.* Zenodo. https://doi.org/10.5281/zenodo.5148500
